# Supplementary material for: Synthesis of Flavonol-Bearing Probes for Chemoproteomic and Bioinformatic Analyses of Asteraceae Petals in Search of Novel Flavonoid Enzymes
Source: Int J Mol Sci. 2023 Jun 3;24(11):9724. doi: 10.3390/ijms24119724 (PMC10253404; doi:10.3390/ijms24119724)
Supplement: Supplementary file 1 [file ijms-24-09724-s001.zip › HigherFlavo - SI.pdf]

Synthesis of Flavonol-Bearing Probes for Chemoproteomic and Bioinformatic Analyses of Asteraceae Petals in Search of Novel Flavonoid Enzymes

Karl Kempf, Oxana Kempf, Yoan Capello, Christian Molitor, Claire Lescoat, Rana Melhem, Stéphane Chaignepain, Elisabeth Génot, Alexis Groppi, Macha Nikolski, Heidi Halbwirth, Denis Deffieux,* and Stéphane Quideau*

SUPPLEMENTARY MATERIALS

Table of Contents

I. Chemical synthesis **S2**

I.1 General **S2**

I.2 Synthesis of quercetin-bearing probes Q6, Q8 and kaempferol-bearing probe K8 .... **S3**

II. Chemoproteomics **S13**

II.1 Reagents and buffers **S13**

II.2 DCPIP titration **S13**

III. Bioinformatics **S14**

IV. NMR Spectra **S17**

Annexes **S38**

I. Chemical synthesis

I.1 General

All reactions were carried out under a nitrogen or argon atmosphere using dry solvents under anhydrous conditions, unless otherwise noted. Solvents for solution phase reactions were either dispensed from a solvent purification system that passes solvents through packed columns of dry neutral alumina [dichloromethane (DCM), toluene] or purified by distillation from sodium/benzophenone [tetrahydrofuran (THF)] under argon immediately before use. *N,N'*-dimethylformamide (DMF) was purchased in anhydrous grade and used without further purification. Methanol (MeOH) was distilled from CaH_2 under argon. Solvents for chromatographic purifications, *i.e.,* ethyl acetate (EtOAc), petroleum ether (PE) and cyclohexane (CH) were purchased at the highest commercial quality. HPLC grade acetonitrile (CH_3CN) and MilliQ water were used for HPLC analyses and purifications. Reagents were purchased at the highest commercial quality and used without further purification unless otherwise stated. Evaporations were conducted under reduced pressure at 35°C unless otherwise noted. Reactions were monitored by analytical thin layer chromatography (TLC) carried out on 0.25 mm Merck silica gel 60 F254 plates. Compounds were visualized with a UV lamp (λ 254, 365 nm) and stained with a solution of potassium permanganate. For highly polar compounds, reactions were monitored by HPLC on a Thermo Spectra system equipped with P1000 XR pumps and a UV 6000 LP diode array detector using reverse-phase Pyramid C18 column (4.6 × 250 mm, 5 µm), and MeCN/H_2O + 0.1% HCOOH at 1 mL/min as the mobile phase (UV detection at 371-375 nm). Flash column chromatography was carried out under positive pressure using Merck silica gel (60, 40-63 µm) and the indicated solvents or was performed on a puriFlash® Interchim system using the indicated solvents and cartridge columns. IR spectra were recorded on a Bruker IFS55 FT-IR spectrometer. NMR spectra of samples in the indicated solvent were recorded on Bruker Avance 300, 400 or 600 MHz spectrometer and were calibrated using residual nondeuterated solvent as internal reference ($CHCl_3$ = 7.26/77.16, DMSO = 2.50/39.52 ppm, MeOH = 3.31/49.0). The following abbreviations were used to indicate multiplicities: s = singlet, d = doublet, t = triplet, m = multiplet, bs = broad singlet. Carbon multiplicities were determined by DEPT 135 experiments. Diagnostic correlations were obtained by two-dimensional COSY, HSQC and HMBC experiments. High resolution (HRMS) mass spectrometric analyses were obtained by electrospray ionization (ESI) and performed at the Centre d'Etude Structurale et d'Analyse des Molécules Organiques (CESAMO) of the Institut des Sciences Moléculaires (ISM, CNRS-UMR 5255, Talence, France).

General Procedure A – Heck coupling.

A mixture of ethyl acrylate (10 equiv.), $Pd(OAc)_2$ (0.2-0.5 equiv.), K_2CO_3 (2.5 equiv.) and iodide **4** (**a**, **b** or **c**, 1 equiv.) in DMF (6 mL) was degassed, flushed with argon and heated at 90 °C for 20 h in a sealed tube [20]. The reaction mixture was cooled to room temperature and partitioned between saturated aqueous NH_4Cl (20 mL) and EtOAc (50 mL). After separation, the aqueous layer was re-extracted twice with EtOAc (2 × 50 mL). The combined organic layers were washed with H_2O (20 mL) and brine (20 mL), dried over Na_2SO_4, filtered and evaporated. The resulting crude product was purified using the puriFlash® Interchim system equipped with a PF-50SIHP/25G column.

General Procedure B – Hydrogenolytic benzyl ether deprotection and regioselective olefin hydrogenation.

Compound **5** (**a**, **b** or **c**, 1 equiv.) was dissolved in DCM, then THF and MeOH were added (DCM/THF/MeOH 1:4:4, v/v/v, 9-36 mL). The final solution was degassed and flushed with nitrogen. Palladium on carbon (10 wt. %, *ca* 1 equiv. of Pd) [21] was added under nitrogen and the mixture was then stirred at room temperature under an H_2 atmosphere (balloon) for 22 h, after which time HPLC monitoring indicated the completion of the reaction. The reaction mixture was filtered through Celite®, which was washed with methanol, and the filtrate was evaporated to give a crude product that was then used without chromatographic purification.

General Procedure C – Lactonization.

The product prepared through the general procedure B (1 equiv.) was suspended in toluene (5 mL) and this suspension was degassed. Trifluoroacetic acid (TFA, 2% v/v) was added under argon, and the reaction mixture was heated at 110 °C for 2 h in a sealed tube. After evaporation, the crude product was directly used without chromatographic purification.

General Procedure D – Final coupling

Compound **6** (**a**, **b** or **c**,1 equiv.) was dissolved in anhydrous DMF (4 mL), degassed and flushed with argon, and the biotinylated amine **7** (1.1 equiv.) [15,16] was added. The resulting mixture was stirred under argon at room temperature for 45 min to 1 h, the advancement of the reaction being monitored by HPLC analysis. The resulting crude product was purified using the puriFlash® Interchim system equipped with a PF-30C18AQ-F0025 column, eluting with 30-40% CH_3CN, 60-70% H_2O + 0.1% HCOOH) at 15 mL/min.

I.2 Synthesis of quercetin-bearing probes Q6, Q8 and kaempferol-bearing probe K8

Compound 3a (2-(3,4-bis(benzyloxy)phenyl)-3,5,7-tris(benzyloxy)-4H-chromen-4-one)

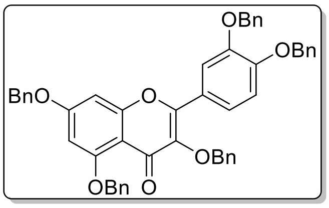

To a solution of quercetin (1 g, 3.31 mmol) in DMF (20 mL) was added K_2CO_3 (6.91 g, 50 mmol) followed by dropwise addition of benzyl bromide (5.1 mL, 43 mmol) at room temperature. The reaction mixture was heated at 70 °C for 15 h under stirring, and after cooling to room temperature, 30 mL of water was added and stirring was continued for an additional hour. The precipitated solid was filtered, washed with water and recrystallized from EtOAc to furnish the known pentabenzylated quercetin **3a** [17] as a white solid (**R***f* = 0.28 [CH/EtOAc (4:1)], 1.77 g, 71%).

^{1}H-NMR (300 MHz, $CDCl_3$) δ ppm: 4.97 (s, 2 H) 5.09 (s, 2 H) 5.15 (s, 2 H) 5.23 (s, 2 H) 5.25 (s, 2 H) 6.45 (d, J=1.5 Hz, 1 H) 6.54 (d, J=1.5 Hz, 1 H) 6.96 (d, J=8.6 Hz, 1 H) 7.25 - 7.52 (m, 23 H) 7.58 (dd, J=8.6, 1.5 Hz, 1 H) 7.66 (d, J=7.3 Hz, 2 H) 7.80 (d, J=1.5 Hz, 1 H).

HRMS (pos. ESI) calcd for $C_{50}H_{41}O_7^+$ $[M+H]^+$ 753.28468, found 753.28392.

Spectroscopic and characteristic data of compound **3a** are consistent with those previously reported [17].

Compound 3b (2-(3,4-bis(benzyloxy)phenyl)-3,7-bis(benzyloxy)-5-hydroxy-4H-chromen-4-one)

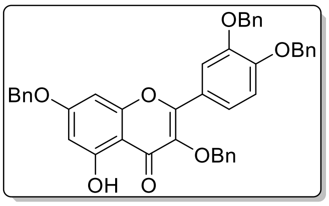

To a stirred solution of quercetin (1 g, 3.31 mmol) in DMF (20 mL) was added K_2CO_3 (2.29 g, 16.55 mmol) and benzyl bromide (2.36 mL, 19.86 mmol). The mixture was stirred at room temperature for 10 h, after which time it was diluted with DCM (20 mL) and poured into aqueous HCl (0.1 M) (10 mL). The organic layer was separated, washed with H_2O (3 × 10 mL), dried over Na_2SO_4, filtered and evaporated. The resulting crude product was purified by flash column chromatography, eluting with PE/EtOAc (4:1), to afford the known tetrabenzylated quercetin compound **3b** [17] as a yellow solid (**R***f* = 0.49 [CH/EtOAc (4:1)], 1.5 g, 68%).

^{1}H-NMR (300 MHz, $CDCl_3$) δ ppm: 4.99 (s, 2 H) 5.04 (s, 2 H) 5.13 (s, 2 H) 5.25 (s, 2 H) 6.45 (dd, J=7.7, 2.0 Hz, 2 H) 6.96 (d, J=8.7 Hz, 1 H) 7.22 - 7.49 (m, 20 H) 7.55 (dd, J=8.7, 2.0 Hz, 2 H) 7.71 (d, J=2.0 Hz, 1 H).

HRMS (pos. ESI) calcd for $C_{43}H_{34}O_7Na^+$ $[M+Na]^+$ 685.21967, found 685.21937.

Spectroscopic and characteristic data of compound **3b** are consistent with those previously reported [17].

Compound 3c (3,5,7-tris(benzyloxy)-2-(4-(benzyloxy)phenyl)-4H-chromen-4-one)

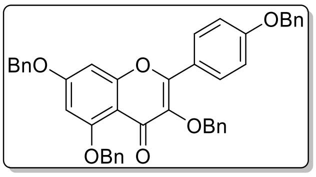

To a solution of kaempferol (423 mg, 1.5 mmol) in DMF (2 mL) was added K_2CO_3 (1.035 g, 7.5 mmol) followed by dropwise addition of benzyl bromide (0.89 mL, 7.5 mmol) at room temperature. The reaction mixture was stirred at room temperature for 24 h, after which time it was then diluted with EtOAc (20 mL) and poured into aqueous HCl (1 M) (10 mL). The organic layer was separated, washed with saturated aqueous $NaHCO_3$ (20 mL) and brine (20 mL), dried over Na_2SO_4, filtered and evaporated. The resulting crude product was purified by flash column chromatography, eluting with PE/DCM/EtOAc (15:4:1), to afford the known tetrabenzylated kaempferol compound **3c** [23] as a white solid (**R***f* = 0.49 [CH/EtOAc (3:1)], 468 mg, 48%) and a tribenzylated kaempferol by-product as a yellow solid (38%).

^{1}H-NMR (300 MHz, $CDCl_3$) δ ppm: 5.09 (s, 2H), 5.10 (s, 2H), 5.14 (s, 2H), 5.29 (s, 2H), 6.52 (dd, J = 32.3, 2.2 Hz, 2H), 7.01 (d, J = 9.0 Hz, 2H), 7.20 – 7.65 (m, 20H), 7.97 (d, J = 9.0 Hz, 2H).

HRMS (pos. ESI) calcd for $C_{43}H_{35}O_6^+$ $[M+H]^+$ 647.24282, found 647.24127.

Spectroscopic and characteristic data of compound **3c** are consistent with those previously reported [22].

Compound 4a (2-(3,4-bis(benzyloxy)phenyl)-3,5,7-tris(benzyloxy)-8-iodo-4H-chromen-4-one)

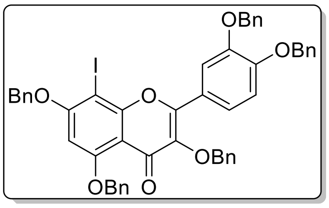

To a solution of **3a** (440 mg, 0.58 mmol) in dry DCM (40 mL) were added *N*-iodosuccinimide (NIS,165 mg, 0.725 mmol) and silver(I) bis(trifluoromethanesulfonyl)imide ($AgNTF_2$, 50 mg, 0.12 mmol) under argon and protected from light. This reaction mixture was stirred at room temperature for 20 h, after which time it was quenched by adding a mixture of aqueous $Na_2S_2O_3$ (4 mL), saturated aqueous $NaHCO_3$ (15 mL) and H_2O (15 mL), and then extracted with DCM (2 × 50 mL). The organic layers were washed with brine (20 mL), dried over Na_2SO_4, filtered and evaporated. The resulting crude product was purified by flash column chromatography, eluting with PE/DCM/EtOAc (75:20:5) to remove some impurities, and then with DCM/EtOAc (4:1), to afford the desired product **4a** as a slightly yellow solid (**R***f* = 0.31 [CH/EtOAc (3:1)], 474 mg, 93%).

^{1}H-NMR (300 MHz, $CDCl_3$) δ ppm: 5.04 (s, 2 H) 5.13 (s, 4 H) 5.24 (s, 2 H) 5.28 (s, 2 H) 6.42 (s, 1 H) 6.99 (d, J=8.6 Hz, 1 H) 7.26 - 7.50 (m, 23 H) 7.56 (d, J=7.2 Hz, 2 H) 7.95 (dd, J=8.6, 2.0 Hz, 1 H) 8.00 (d, J=2.0 Hz, 1 H).

HRMS (pos. ESI) calcd for $C_{50}H_{40}O_7I^+$ $[M+H]^+$ 879.18132, found 879.18078.

Spectroscopic and characteristic data of compound **4a** are consistent with those previously reported [19].

Compound 4b (2-(3,4-bis(benzyloxy)phenyl)-3,7-bis(benzyloxy)-5-hydroxy-6-iodo-4H-chromen-4-one)

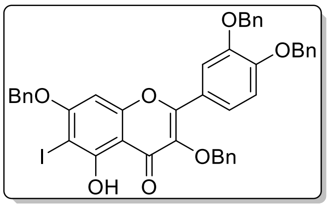

To a solution of **3b** (810 mg, 1.22 mmol) in dry DCM (40 mL) was added NIS (330 mg, 1.46 mmol) under nitrogen and protected from light. This reaction mixture was stirred at room temperature for 20 h, after which time it was diluted with DCM (20 mL) and saturated aqueous $NaHCO_3$ was added (40 mL). Following extraction with DCM (3 × 40 mL), the combined organic layers were washed with H_2O (40 mL), brine (40

mL), dried over Na_2SO_4, filtered and evaporated. The resulting crude product was purified by flash column chromatography, eluting with PE/DCM/EtOAc (75:20:5) to remove some impurities, and then with DCM/EtOAc (4:1), to afford the desired product **4b** as pale yellow solid (**R***f* = 0.55 [CH/EtOAc (4:1)], 820 mg, 85%).

^{1}H-NMR (300 MHz, $CDCl_3$) *δ* ppm: 5.00 (s, 2H), 5.05 (s, 2H), 5.25 (s, 4H), 6.47 (s, 1H), 6.96 (d, *J* = 8.7 Hz, 1H), 7.21 – 7.25 (m, 4H), 7.30 – 7.56 (m, 17H), 7.69 (s, 1H), 13.72 (s, 1H).

HRMS (pos. ESI) calcd for $C_{43}H_{34}O_7I^+$ $[M+H]^+$ 789.13437, found 789.13408.

Spectroscopic and characteristic data of compound **4b** are consistent with those previously reported [19].

Compound 4c (3,5,7-tris(benzyloxy)-2-(4-(benzyloxy)phenyl)-8-iodo-4H-chromen-4-one)

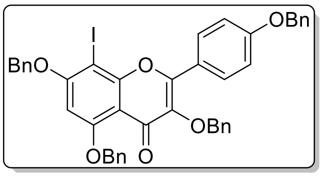

To a solution of **3c** (70 mg, 0.11 mmol) in dry DCM (10 mL) were added NIS (31 mg, 0.14 mmol) and $AgNTF_2$ (8.5 mg, 0.022 mmol) under argon and protected from light. This reaction mixture was stirred at room temperature for 20 h, after which time it was quenched by adding a mixture of aqueous $Na_2S_2O_3$ (1 mL), saturated aqueous $NaHCO_3$ (5 mL) and H_2O (5 mL), and then extracted with DCM (2 × 20 mL). The organic layers were washed with brine (10 mL), dried over Na_2SO_4, filtered and evaporated. The resulting crude product was purified by flash column chromatography, eluting with PE/DCM/EtOAc (75:20:5), to afford the desired product **4c** as a slightly yellow solid (**R***f* = 0.39 [Cy/EtOAc (3:1)], 85 mg, 100%).

IR ν_{max} 3057, 2956, 2918, 2851, 2305, 1733, 1634, 1593, 1508, 1454, 1401, 1380, 1336, 1256, 1183, 1109, 895, 936, 734, 696 cm^{-1}.

^{1}H-NMR (300 MHz, $CDCl_3$) *δ* ppm: 5.12 (s, 2H), 5.14 (s, 4H), 5.29 (s, 2H), 6.43 (s, 1H), 7.04 (d, *J* = 9.1 Hz, 2H), 7.28 – 7.59 (m, 20H), 8.27 (d, *J* = 9.0 Hz, 2H).

^{13}C-NMR (75 MHz, $CDCl_3$) *δ* ppm: 173.6, 161.4, 160.6, 160.6, 156.7, 154.0, 139.5, 137.0, 136.6, 136.3, 135.6, 130.9, 130.9, 129.0, 129.0, 128.9, 128.9, 128.9, 128.9, 128.8, 128.8, 128.4, 128.3, 128.3, 128.3, 128.2, 128.0, 127.6, 127.6, 127.1, 127.1, 126.9, 126.9, 123.5, 114.8, 114.8, 110.8, 95.8, 74.1, 71.5, 71.4, 70.2, 66.2.

HRMS (pos. ESI) calcd for $C_{43}H_{34}O_6I^+$ $[M+H]^+$ 773.13946, found 773.13721.

Compound 5a ((2E)-ethyl 3-(2-(3,4-bis(benzyloxy)phenyl)-3,5,7-tris(benzyloxy)-4-oxo-4H-chromen-8-yl) acrylate)

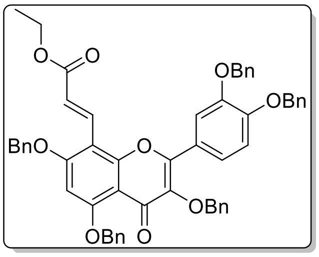

This Heck coupling-based preparation was performed according to the general procedure A: ethyl acrylate (300 µL, 2.75 mmol), $Pd(OAc)_2$ (12 mg, 0.055 mmol), K_2CO_3 (95 mg, 0.69 mmol), iodide **4a** (242 mg, 0.275 mmol). The resulting crude product was purified using the puriFlash® Interchim system, first eluting with PE/DCM/EtOAc (50:20:5) to remove some impurities and then with PE/DCM/EtOAc (75:40:10), to afford the desired new product **5a** as a white solid (**R***f* = 0.42 [CH/EtOAc (3:1)], 109 mg, 87%).

IR ν_{max} 3032, 2927, 1729, 1704, 1621, 1591, 1511, 1499, 1454, 1414, 1382, 1345, 1319, 1300, 1269, 1200, 1170, 1104, 1024, 911, 857, 805, 735, 696, 644, 626 cm^{-1}.

^{1}H-NMR (300 MHz, $CDCl_3$) *δ* ppm: 1.27 (t, J=7.2 Hz, 3 H, OCH_2CH_3) 4.20 (q, J=7.2 Hz, 2 H, OCH_2CH_3) 5.06 (s, 2 H, Bn) 5.11 (s, 2 H, Bn) 5.19 (s, 2 H, Bn) 5.24 (s, 2 H, Bn) 5.26 (s, 2 H, Bn) 6.42 - 6.46 (m, 1 H, 6) 6.88 (d, J=16.1 Hz, 1 H, CH*CH*COOEt) 6.99 (d, J=8.4 Hz, 1 H, 5') 7.24 (s, 4 H, Bn) 7.31 - 7.43 (m, 16 H,

Bn) 7.44 - 7.56 (m, 5 H, Bn) 7.68 (dd, J=8.4, 2.0 Hz, 1 H, 6’) 7.79 (d, J=2.0 Hz, 1 H, 2’) 8.28 (d, J=16.1 Hz, 1 H, *CH*CHCOOEt).

^{13}C-NMR (75 MHz, $CDCl_3$) δ ppm: 173.8, 168.1, 161.9, 160.8, 156.5, 153.5, 150.7, 148.6, 139.7, 137.2, 137.0, 136.9, 136.1, 135.5, 133.3, 128.9, 128.9, 128.9, 128.8, 128.8, 128.6, 128.6, 128.6, 128.5, 128.4, 128.4, 128.3, 128.3, 128.1, 128.0, 128.0, 127.8, 127.4, 127.4, 127.2, 127.2, 127.1, 127.1, 126.7, 126.7, 123.7, 122.4, 120.8, 114.3, 113.9, 109.9, 105.4, 95.0, 74.1, 71.1, 71.1, 70.9, 70.7, 60.3, 14.4.

HRMS (pos. ESI) calcd for $C_{55}H_{47}O_9^+$ $[M+H]^+$ 851.32146, found 851.32073.

Compound 5b ((2E)-ethyl 3-(2-(3,4-bis(benzyloxy)phenyl)-3,7-bis(benzyloxy)-5-hydroxy-4-oxo-4H-chromen-6-yl)acrylate)

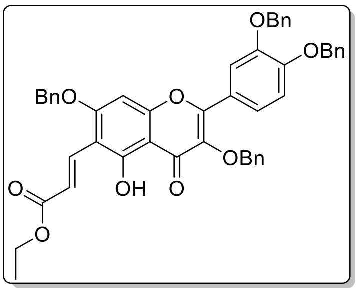

This Heck coupling-based preparation was performed according to the general procedure A: ethyl acrylate (0.218 mL, 2 mmol), $Pd(OAc)_2$ (23 mg, 0.1 mmol), K_2CO_3 (69 mg, 0.5 mmol), iodide **4b** (158 mg, 0.2 mmol). The resulting crude product was purified using the puriFlash® Interchim (column PF-50SIHP/25G, dry package), first eluting with PE/DCM/EtOAc (50:20:5) to remove some impurities and then with PE/DCM/EtOAc (75:40:10), to afford the desired new product **5b** as a yellow solid (**R***f* = 0.44 [CH/EtOAc (3:1)], 65 mg, 43%).

IR ν_{max} 3032, 2924, 1729, 1704, 1643, 1599, 1509, 1480, 1454, 1431, 1371, 1348, 1325, 1305, 1270, 1219, 1198, 1169, 1105, 1018, 911, 860, 805, 735, 695, 641, 624 cm^{-1}.

^{1}H-NMR (300 MHz, $CDCl_3$) δ ppm: 1.32 (t, J=7.2 Hz, 3 H, OCH_2*CH_3*) 4.23 (q, J=7.2 Hz, 2 H, O*CH_2*CH_3) 4.97 (s, 2 H, Bn) 5.04 (s, 2 H, Bn) 5.23 (d, J=5.1 Hz, 4 H, Bn) 6.41 (s, 1 H, 8) 6.95 (d, J=8.6 Hz, 1 H, 5’) 7.04 (d, J=16.3 Hz, 1 H, CH*CH*COOEt) 7.22 - 7.49 (m, 20 H, Bn arH) 7.54 (dd, J=8.6, 1.9 Hz, 1 H, 6’) 7.70 (d, J=1.9 Hz, 1 H, 2’) 8.14 (d, J=16.3 Hz, 1 H, *CH*CHCOOEt) 13.90 (s, 1 H, OH).

^{13}C-NMR (75 MHz, $CDCl_3$**)** δ ppm: 178.9, 168.5, 162.9, 162.2, 157.0, 156.4, 151.4, 148.3, 137.7, 137.0, 136.7, 136.4, 135.5, 133.6, 128.9, 128.9, 128.9, 128.9, 128.7, 128.7, 128.6, 128.6, 128.5, 128.4, 128.4, 128.4, 128.2, 128.0, 127.5, 127.5, 127.3, 127.3, 127.2, 127.2, 123.1, 122.7, 121.1, 115.4, 113.7, 107.5, 105.8, 91.0, 74.5, 71.2, 71.1, 70.9, 60.3, 14.5.

HRMS (pos. ESI) calcd for $C_{48}H_{40}O_9Na^+$ $[M+Na]^+$ 783.25645, found 783.25532.

Compound 5c ethyl (E)-3-(3,5,7-tris(benzyloxy)-2-(4-(benzyloxy)phenyl)-4-oxo-4H-chromen-8-yl)acrylate

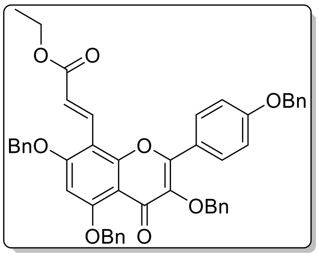

This Heck coupling-based preparation was performed according to the general procedure A: ethyl acrylate (0.173 mL, 1.61 mmol), $Pd(OAc)_2$ (18 mg, 0.08 mmol), K_2CO_3 (51 mg, 0.32 mmol), iodide **4c** (110 mg, 0.16 mmol), DMF (3 mL). The resulting crude product was purified using the puriFlash® Interchim, first eluting with PE/DCM/EtOAc (50:20:5) to remove some impurities and then with CH/DCM/EtOAc (75:40:10), to afford the desired new product **5c** as a white solid (**R***f* = 0.29 [CH/EtOAc (4:1)], 50 mg, 48%).

IR ν_{max} 3062, 2918, 1738, 1702, 1622, 1605, 1587, 1508, 1454, 1415, 1381, 1340, 1296, 1254, 1177, 1123, 1099, 1024, 985, 910, 834, 805, 734, 696, 644, 624 cm^{-1}.

^{1}H-NMR (300 MHz, $CDCl_3$) *δ* ppm: 1.34 (t, *J* = 7.1 Hz, 3H), 4.25 (q, *J* = 7.1 Hz, 2H), 5.12 (s, 2H), 5.16 (s, 2H), 5.20 (s, 2H), 5.29 (s, 2H), 6.46 (s, 1H), 6.88 (d, *J* = 16.3 Hz, 1H), 7.06 (d, *J* = 9.0 Hz, 2H), 7.23 – 7.57 (m, 20H), 8.04 (d, *J* = 9.0 Hz, 2H), 8.25 (d, *J* = 16.3 Hz, 1H).

^{13}C-NMR (75 MHz, $CDCl_3$) *δ* ppm: 174.0, 168.2, 161.9, 160.9, 160.6, 156.6, 154.0, 139.6, 137.0, 136.6, 136.1, 135.5, 133.3, 130.5, 129.0, 129.0, 129.0, 128.9, 128.9, 128.8, 128.8, 128.6, 128.3, 128.3, 128.1, 128.1, 128.1, 127.6, 127.6, 127.2, 127.2, 126.8, 126.8, 123.4, 121.0, 114.9, 110.1, 105.6, 95.2, 77.6, 77.2, 76.7, 74.1, 71.2, 71.2, 70.2, 60.4, 14.5.

HRMS (pos. ESI) calcd for $C_{48}H_{41}O_8^+$ $[M+H]^+$ 745.27959, found 745.27928.

Compound 6a

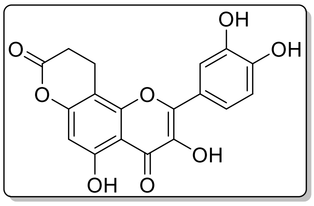

Step 1. The debenzylation and the regioselective olefin hydrogenation of **5a** was performed according to the general procedure B: **5a** (194 mg, 0.228 mmol), (DCM/THF/MeOH 1:4:4, v/v/v, 36 mL), 10 wt. % Pd/C (240 mg, 0.228 mmol Pd). The resulting crude product was obtained as a yellow solid (93 mg) (**HPLC analysis:** t_R = 8 min (λ_{max} = 375 nm), 40% CH_3CN/60% H_2O + 0.1% HCOOH).

HRMS (neg. ESI) calcd for $C_{20}H_{17}O_9^-$ $[M-H]^-$ 401.08781, found 401.08819.

Step 2. Lactonization was performed according to the general procedure C: crude product from step 1 (15 mg, 0.04 mmol), toluene (5 mL), TFA (100 µL, 2% v/v). The resulting new product **6a** was obtained as a yellow solid (14 mg) (**HPLC analysis:** t_R = 9 min (λ_{max} = 375 nm), 40% CH_3CN/60 % H_2O + 0.1% HCOOH).

IR ν_{max} 3350, 2923, 2853, 1740, 1649, 1625, 1557, 1513, 1354, 1315, 1261, 1136, 1079, 960, 889, 793, 733 cm^{-1}.

^{1}H-NMR (400 MHz, DMSO-*d*$_6$) *δ* ppm: 2.90 (t, *J*=7.3 Hz, 2 H) 3.15 (t, *J*=7.3 Hz, 2 H) 6.47 (s, 1 H) 6.90 (d, *J*=8.6 Hz, 1 H) 7.60 (dd, *J*=8.6, 1.7 Hz, 1 H) 7.71 (d, *J*=1.7 Hz, 1 H) 9.29 - 9.82 (m, 3 H) 12.45 (br. s, 1 H).

^{13}C-NMR (100 MHz, DMSO-*d*$_6$) *δ* ppm: δ 176.4, 167.4, 158.9, 156.1, 151.7, 148.3, 147.9, 145.4, 136.6, 121.9, 120.5, 115.9, 115.2, 106.2, 101.8, 98.6, 27.8, 16.6.

HRMS (neg. ESI) calcd for $C_{18}H_{11}O_8^-$ $[M-H]^-$ 355.04594, found 355.04607.

Compound 6b

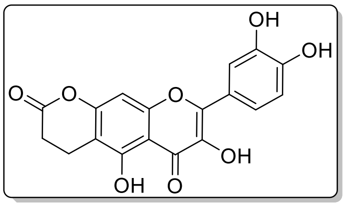

Step 1. The debenzylation and the regioselective olefin hydrogenation of **5b** was performed according to the general procedure B: **5b** (130 mg, 0.171 mmol), (DCM/THF/MeOH 1:4:4, v/v/v, 25 mL), 10 wt. % Pd/C (182 mg, 0.171 mmol Pd). The resulting crude product was obtained as a yellow solid (68 mg) (**HPLC analysis:** t_R = 11 min (λ_{max} = 369 nm), 40% CH_3CN/60 % H_2O + 0.1% HCOOH).

HRMS (pos. ESI) calcd for $C_{20}H_{18}O_9Na^+$ $[M+Na]^+$ 425.08430, found 425.08375.

Step 2. Lactonization was performed according to the general procedure C: crude product from step 1 (19 mg, 0.047 mmol), toluene (5 mL), TFA (120 µL, 2% v/v). The resulting new product **6b** was obtained as a yellow solid (17 mg) (**HPLC analysis:** t_R = 9 min (λ_{max} = 374 nm), 40% CH_3CN/60 % H_2O + 0.1% HCOOH).

IR ν_{max} 3351, 2923, 2853, 1740, 1650, 1625, 1557, 1513, 1355, 1315, 1261, 1136, 1080, 960, 890, 793, 733 cm^{-1}.

^{1}H-NMR (300 MHz, DMSO-*d*$_6$) *δ* ppm: 2.82 – 2.88 (m, 2H), 2.91 – 2.98 (m, 2H), 6.89 (s, 1H), 6.92 (s, 1H), 7.58 (dd, J = 8.5, 2.1 Hz, 1H), 7.74 (d, J = 2.1 Hz, 1H), 9.33 (s, 1H), 9.63 (s, 1H), 9.69 (s, 1H), 12.96 (s, 1H).

^{13}C-NMR (75 MHz, DMSO-*d*$_6$) *δ* ppm: 176.1, 167.3, 156.4, 156.3, 153.7, 148.3, 148.1, 145.1, 136.2, 121.6, 120.3, 115.6, 115.4, 105.7, 105.0, 95.0, 27.6, 16.2.

HRMS (neg. ESI, in methanol**)** calcd for $C_{19}H_{15}O_8^-$ [MOMe-H]$^-$ 371.07724, found 371.07665.

Compound 6c

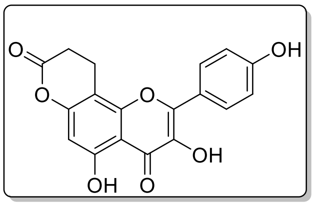

Step 1. The debenzylation and the regioselective olefin hydrogenation of **5c** was performed according to the general procedure B: **5c** (40 mg, 0.054 mmol), (DCM/THF/MeOH 1:4:4, v/v/v, 9 mL), 10 wt. % Pd/C (58 mg, 0.054 mmol Pd). The resulting crude product was obtained as a yellow solid (21 mg) (**HPLC analysis:** t_R = 3.8 min (λ_{max} = 371 nm), 70% CH_3CN/30% H_2O + 0.1% HCOOH).

Step 2. Lactonization was performed according to the general procedure C: crude product from step 1 (18 mg, 0.047 mmol), toluene (5 mL), TFA (120 µL, 2% v/v). The resulting new product **6c** was obtained as a yellow solid (16 mg) (**HPLC analysis:** t_R = 7.8 min (λ_{max}=371 nm), 40% CH_3CN/60 % H_2O + 0.1% HCOOH).

IR ν_{max} 3350, 2923, 2853, 1745, 1650, 1613, 1574, 1515, 1358, 1261, 1137, 845, 825, 762 cm^{-1}.

^{1}H-NMR (400 MHz, DMSO-*d*$_6$) *δ* ppm: 2.91 (t, *J* = 7.4 Hz, 2H), 3.17 (t, *J* = 7.4 Hz, 2H), 6.51 (s, 1H), 6.95 (d, *J* = 8.9 Hz, 2H), 8.09 (d, *J* = 8.9 Hz, 2H), 9.71 (s, 1H), 10.21 (s, 1H), 12.47 (s, 1H).

^{13}C-NMR (100 MHz, DMSO-*d*$_6$) *δ* ppm: 176.3, 167.2, 159.6, 158.8, 156.0, 151.5, 147.8, 136.4, 129.6, 129.6, 121.4, 115.6, 115.6, 106.1, 101.7, 98.5, 27.6, 16.4.

HRMS (neg. ESI) calcd for $C_{18}H_{11}O_7^-$ [M-H]$^-$ 339.04993, found 339.05067.

Probe Q8

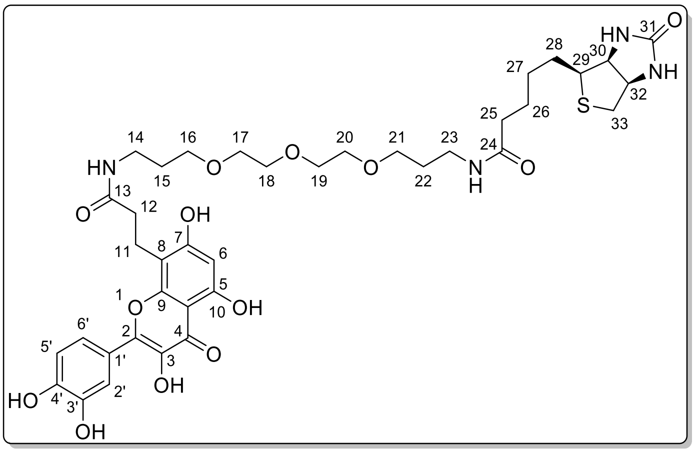

The **final coupling** was performed according to the general procedure D: **6a** (10 mg, 0.028 mmol), DMF (4 mL), **7** (26 mg, 0.058 mmol). The resulting crude product was purified twice using the puriFlash® Interchim system, eluting with 40% CH_3CN/60 % H_2O + 0.1% HCOOH, isocratic mode) to afford the probe **Q8** as a yellow solid (11 mg, 49%) (**HPLC analysis:** t_R = 3.6 min (λ_{max} = 375 nm), 40% CH_3CN/60% H_2O + 0.1% HCOOH).

IR ν_{max} 3349, 2918, 2850, 1686, 1647, 1560, 1464, 1374, 1262, 1201, 1070, 961, 736 cm^{-1}.

^{1}H-NMR (600 MHz, methanol-*d*$_4$) *δ* ppm: 1.39 (quintet, *J* = 7.7 Hz, 2 H) 1.56 - 1.75 (m, 8 H) 2.16 (t, *J* = 7.3 Hz, 2 H) 2.53 (t, *J* = 7.2 Hz, 2 H) 2.68 (d, *J* = 12.6 Hz, 1 H) 2.89 (dd, *J* = 12.6, 5.1 Hz, 1 H) 3.14 (t, *J* = 7.2 Hz, 2 H), 3.15-3.18 (m, 1H), 3.18 (t, *J* = 6.6 Hz, 2 H) 3.23 (t, *J* = 6.8 Hz, 2 H) 3.34 (t, *J* = 6.1 Hz, 2 H), 3.44 - 3.50 (m, 4 H), 3.51 - 3.54 (m, 2 H), 3.54 - 3.58 (m, 4 H), 4.26 (dd, *J*=7.3, 4.4 Hz, 1 H), 4.46 (dd, *J* = 7.7, 5.1), 6.21 (s, 1 H) 6.90 (d, *J* = 8.4 Hz, 1 H) 7.74 (d, *J* = 8.4 Hz, 1 H) 7.81 (s, 1 H).

^{13}C-NMR (150 MHz, methanol-d_4) δ ppm: 177.4, 176.0, 175.8, 166.1, 165.3, 160.5, 155.6, 148.9, 147.6,146.4, 137.1, 124.4, 121.9, 116.4, 115.7, 106.7, 104.0, 99.7, 71.4, 71.4, 71.1, 71.1, 69.9, 69.8, 63.4, 61.6, 57.0, 41.0, 37.8, 37.8, 36.8, 36.6, 30.4, 30.2, 29.8, 29.5, 26.9, 20.2.

HRMS (neg. ESI) calcd for $C_{38}H_{49}O_{13}N_4S^-$ [M-H]$^-$ 801.30113, found 801.30184.

Position	δ_H (mult., J in Hz)	δ_C	COSY	HSQC	HMBC
**Quercetin moiety**					
2	-	147.6	-	-	C2', C6'
3	-	137.1	-	-	-
5	-	160.5	-	-	H6
6	6.2 (s)	99.7	-	C6	C5, C7, C8, C10
7	-	165.3	-	-	H6, H11
8	-	106.7	-	-	H6, H11, H12
9	-	155.6	-	-	H11
10	-	104.0	-	-	H6
1'	-	124.4	-	-	H2', H5'
2'	7.81 (s)	115.7	-	C2'	H6', C5'
3'	-	146.4	-	-	C2', C5'
4'	-	148.9	-	-	C5'
5'	6.90 (d, 8.4)	116.4	H6'	C5'	C1', C3', C4'
6'	7.74 (d, 8.4)	121.9	H5'	C6'	H2', C2', C2
**Biotin-linker moiety**					
11	3.14 (t, 7.2)	20.2	H12	C11	H12, C7, C8, C9, C12, C13
12	2.53 (t, 7.2)	36.7	H11	C12	H11, C8, C11, C13
14	3.18 (t, 6.6)	37.8	H15	C14	H15, H16, C13, C15, C16
15	1.56 - 1.75 (m)	30.2	H16	C15	H14, H16, C13, C14, C16
16	3.34 (t, 6.1)	69.8	H15	C 16	H14, H15, C14, C15
17-20	3.44 - 3.58 (m)	71.1, 71.1 71.4, 71.4	H17-18, H19-20, H21-22	C17-20	H17-18, H19-20, C17-18, C19-20
21	3.45-3.50 (m)	69.9	H22	C21	H22, H23, C22, C23
22	1.56 - 1.75 (m)	30.4	H21, H23	C22	H21, H23, C21, C23, C24
23	3.23 (t, 6.8)	37.8	H 22	C23	H21, H22, C21, C22, C24
25	2.16 (t, 7.3)	36.8	H26	C25	H26, H27, C24, C26, C28
26	1.56 - 1.75 (m)	26.9	H25	C26	H25, H27, H28, C24, C29
27	1.39 (quin, 7.7)	29.5	H28	C27	H28, C25, C26, C28, C29
28	1.56 - 1.75 (m)	29.8	H27	C28	H25, H27, C26, C27, C29, C30
29	3.15-3.18 (m)	57.0	H28, H30	C29	H26, H27, H28, C30, C32, C33a
30	4.26 (dd, 7.3, 4.4)	63.4	H29, H32	C30	H28, C29, C31, C33, C33a
32	4.46 (dd, *J*=7.7, 5.1)	61.6	H30, H33b	C32	H33a, C29, C31, C33a
33	a : 2.68 (d 12.8)<br>b : 2.89 (dd 12.8, 5.1)	41.0	H33b<br>H32, H33a	C33	H30, H32, C30, C32<br>H30, H32,
**Carbonyls**					
C_4=O	-	177.4	-	-	
C_{13}=O	-	175.8	-	-	H11, H12, H14, H15
C_{24}=O	-	176.0	-	-	H22, H23, H25, H26
C_{31}=O	-	166.1	-	-	H32

Probe Q6

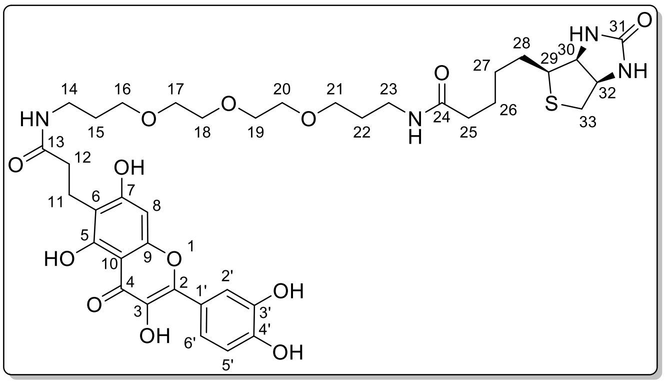

The **final coupling** was performed according to the general procedure D: **6b** (15 mg, 0.045 mmol), DMF (4 mL), **7** (25 mg, 0.05 mmol). The resulting crude product was purified using the puriFlash® Interchim system, eluting with 40% CH_3CN/60 % H_2O + 0.1% HCOOH) to afford the probe **Q6** as a yellow solid (12 mg, 33%) (**HPLC analysis:** t_R = 3.9 min (λ_{max} = 371 nm), 40% CH_3CN/60 % H_2O + 0.1% HCOOH).

IR ν_{max} 3321, 2921, 2853, 1686, 1640, 1564, 1466, 1359, 1266, 1201, 1092, 818 cm^{-1}.

^{1}H NMR (400 MHz, methanol-d_4) δ ppm: 1.40 (q, J = 7.6 Hz, 2H), 1.53 – 1.77 (m, 9H), 2.17 (t, J = 7.4 Hz, 2H), 2.48 (t, J = 7.4 Hz, 2H), 2.68 (d, J = 12.7 Hz, 1H), 2.89 (dd, J = 12.7, 5.0 Hz, 1H), 2.94 (t, J = 7.4 Hz, 2H), 3.10 – 3.18 (m, 1H), 3.20 – 3.24 (m, 4H), 3.41 (t, J = 6.2 Hz, 2H), 3.45 – 3.60 (m, 12H), 4.26 (dd, J = 7.8, 4.4 Hz, 1H), 4.45 (dd, J = 7.9, 5.0 Hz, 1H), 6.37 (s, 1H), 6.87 (d, J = 8.4 Hz, 1H), 7.62 (dd, J = 8.4, 1.5 Hz, 1H), 7.72 (s, 1H).

^{13}C NMR (100 MHz, methanol-d_4) δ ppm: 176.9, 176.1, 176.0, 167.1, 166.1, 159.3, 156.8, 149.1, 147.3, 146.4, 137.0, 124.1, 121.5, 116.3, 115.7, 111.8, 103.3, 94.8, 71.4, 71.4, 71.2, 71.2, 69.9, 69.9, 63.4, 61.6, 57.0, 41.0, 37.9, 37.8, 36.9, 36.3, 30.4, 30.3, 29.8, 29.5, 26.9, 19.8.

HRMS (pos. ESI) calcd for $C_{38}H_{50}O_{13}N_4SNa^+$ $[M+Na]^+$ 825.29873, found 825.29800.

Position	δ_H (mult., J in Hz)	δ_C	COSY	HSQC	HMBC
**Quercetin moiety**					
2	-	147.3	-	-	C2', C6'
3	-	137.0	-	-	-
5	-	159.3	-	-	-
6	-	111.8	-	-	-
7	-	166.1	-	-	-
8	6.37 (s)	94.8	-	C8	C6, C10
9	-	156.8	-	-	-
10	-	103.3	-	-	-
1'	-	124.1	-	-	-
2'	7.72 (s)	115.7	-	C2'	H6', C4', C6'
3'	-	146.4	-	-	H5'
4'	-	149.1	-	-	H5', H6'
5'	6.87 (d, 8.4)	116.3	H6'	C5'	C3', C1'
6'	7.62 (dd, J = 8.4, 1.5 Hz)	121.5	H5', H2'	C6'	H2', C4'
**Biotin-linker moiety**					
11	2.94 (t, 7.4)	19.8	H12	C11	H12, C7, C8, C9, C12, C13
12	2.48 (t, 7.4)	36.3	H11	C12	H11, C6, C11, C13
14	3.20 – 3.24 (m)	37.8	H15	C14	H15, H16, C13, C15, C16
15	1.56 - 1.75 (m)	30.3	H16	C15	H14, H16, C14, C16
16	3.41 (t, 6.2)	69.9	H15	C 16	H14, H15, C14, C15, C17
17-20	3.45 - 3.60 (m)	71.2, 71.2, 71.4, 71.4	H17-18, H19-20, H21-22	C17-20	H17-18, H19-20, C17-18, C19-20
21	3.45-3.60 (m)	69.9	H22	C21	H22, H23, C22, C23

22	1.56 - 1.75 (m)	30.4	H21, H23	C22	H21, H23, C21, C23, C24
23	3.20 – 3.24 (m)	37.9	H 22	C23	H21, H22, C21, C22, C24
25	2.16 (t, 7.3)	36.9	H26	C25	H26, H27, C26, C28
26	1.56 - 1.75 (m)	26.9	H25	C26	H25, H27, H28, C24, C29
27	1.40 (quin, 7.7)	29.5	H28	C27	H28, C29
28	1.53 - 1.77 (m)	29.8	H27, H25	C28	-
29	3.10-3.18 (m)	57.0	H28, H30	C29	H33a
30	4.26 (dd, 7.3, 4.4)	63.4	H29, H32	C30	H33a
32	4.45 (dd, *J*=7.9, 5.0)	61.6	H30, H33b	C32	H33a, C29, C31, C33a
33	a : 2.68 (d, *J* = 12.7)<br>b : 2.89 (dd, *J* = 12.7, 5.0)	41.0	H33b<br>H32, H33a	C33	C30, C32
**Carbonyls**					
C_4=O	-	176.9	-	-	
C_{13}=O	-	176.0	-	-	H11, H12, H14, H15
C_{24}=O	-	176.1	-	-	
C_{31}=O	-	167.1	-	-	H32

Probe K8

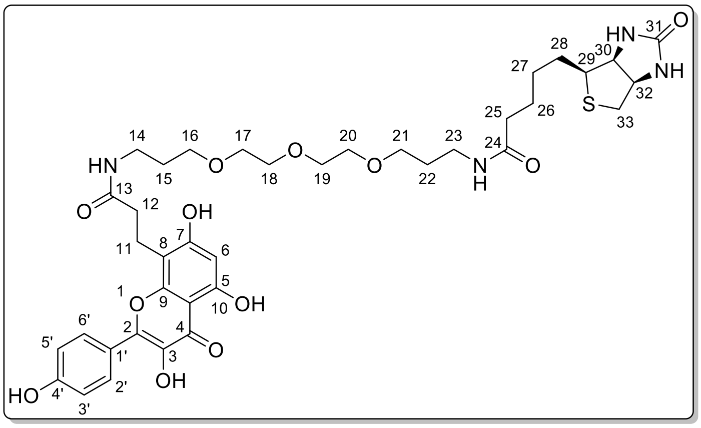

The **final coupling** was performed according to the general procedure D: **6c** (20 mg, 0.058 mmol), DMF (4 mL), **7** (33 mg, 0.074 mmol). The resulting crude product was purified using the puriFlash® Interchim system, eluting with 40% CH_3CN/60 % H_2O + 0.1% HCOOH) to afford the probe **K8** as a yellow solid (13 mg, 29%) (**HPLC analysis:** t_R = 4.6 min (λ_{max}= 372 nm), 40% CH_3CN/60 % H_2O + 0.1% HCOOH).

IR ν_{max} 3331, 2920, 2849, 1686, 1642, 1555, 1450, 1362, 1264, 1176, 841, 733 cm^{-1}.

^{1}H NMR (400 MHz, methanol-d_4) δ ppm: 1.39 (quin, *J* = 7.5 Hz , 2H), 1.50 – 1.80 (m, 9H), 2.17 (t, *J* = 7.3 Hz 2H), 2.51 (t, *J* = 7.0 Hz, 2H), 2.68 (d, *J* = 12.6 Hz, 1H), 2.89 (dd, *J* = 12.6, 5.1 Hz, 1H), 3.13 (t, *J* = 7.0 Hz, 2H), 3.15 – 3.18 (m, 1H), 3.18 (t, *J* = 6.6 Hz, 2H), 3.23 (t, *J* = 6.8 Hz, 2H) 3.36 (t, *J* = 6.1 Hz, 2H), 3.45 – 3.60 (m, 10H), 4.26 (dd, *J* = 7.3, 4.3 Hz, 1H), 4.46 (dd, *J* = 7.3, 4.8 Hz, 1H), 6.23 (s, 1H), 6.93 (d, *J* = 7.9 Hz, 2H), 8.18 (d, *J* = 7.9 Hz, 2H).

^{13}C NMR (100 MHz, methanol-d_4) δ ppm: 177.5, 176.0, 175.7, 166.1, 164.8, 160.6, 160.6, 155.6, 147.8, 137.0, 130.8, 130.8, 124.1, 116.5, 116.5, 106.8, 104.1, 99.6, 71.5, 71.5, 71.2, 71.1, 69.9, 69.8, 63.4, 61.6, 57.0, 41.0, 37.8, 37.8, 36.8, 36.7, 30.4, 30.2, 29.8, 29.5, 26.9, 20.2.

HRMS (pos. ESI) calcd for $C_{38}H_{50}O_{12}N_4SNa^+$ $[M+Na]^+$ 809.30381, found 809.30255.

Position	δ_H (mult., J in Hz)	δ_C	COSY	HSQC	HMBC
**Kaempferol moiety**					
2	-	147.8	-	-	
3	-	137.0	-	-	-
5	-	160.6	-	-	
6	6.23 (s)	99.6	-	C6	
7	-	164.8	-	-	
8	-	106.8	-	-	
9	-	155.6	-	-	
10	-	104.1	-	-	
1’	-	124.1	-	-	H3’, H5’
2’	8.18 (d, 7.9)	130.8	H3’	C2’	C4’
3’	6.93 (d, 7.9)	116.5	H2’	C3’	C1’
4’	-	160.6	-	-	H2’, H6’
5’	6.93 (d, 7.9)	116.5	H6’	C5’	C1’
6’	8.18 (d, 7.9)	130.8	H5’	C6’	C4’
**Biotin-linker moiety**					
11	3.13 (t, 7.0)	20.2	H12	C11	H12, C7, C8, C9, C12, C13
12	2.51 (t, 7.0)	36.7	H11	C12	H11, C8, C11, C13
14	3.18 (t, 6.6)	37.8	H15	C14	H15, H16, C13, C15, C16
15	1.56 - 1.75 (m)	30.2	H16	C15	H14, H16, C13, C14, C16
16	3.36 (t, 6.1)	69.8	H15	C 16	H14, H15, C14, C15
17-20	3.45 - 3.60 (m)	71.1, 71.2 71.5, 71.5	H17-18, H19-20, H21-22	C17-20	H17-18, H19-20, C17-18, C19-20
21	3.45-3.50 (m)	69.9	H22	C21	H22, H23, C22
22	1.56 - 1.75 (m)	30.4	H21, H23	C22	H21, H23, C21, C23, C24
23	3.23 (t, 6.8)	37.8	H 22	C23	H21, H22, C21, C22, C24
25	2.13 (t, 7.3)	36.8	H26	C25	H26, H27, C24, C26
26	1.56 - 1.75 (m)	26.9	H25	C26	H25, H27, H28, C24, C29
27	1.39 (quin, 7.5)	29.5	H28	C27	H28
28	1.56 - 1.75 (m)	29.8	H27	C28	C29, C30
29	3.15-3.18 (m)	57.0	H28, H30	C29	H33a, C33a
30	4.26 (dd, 7.3, 4.4)	63.4	H29, H32	C30	
32	4.46 (dd, *J*=7.7, 5.1)	61.6	H30, H33b	C32	H33a, C29
33	a : 2.68 (d 12.6)<br>b : 2.89 (dd 12.6, 5.1)	41.0	H33b<br>H32, H33a	C33	C30, C32, C29
**Carbonyls**					
C_4=O	-	177.5	-	-	
C_{13}=O	-	175.7	-	-	H11, H12, H14, H15
C_{24}=O	-	176.0	-	-	H22, H23, H25, H26
C_{31}=O	-	166.1	-	-	H32

II. Chemoproteomics

II.1 Reagents and buffers

Capture buffer (12 mM PBS pH 6.8): stored at room temperature in a dark bottle, degassed before use

- 60 mL of 100 mM phosphate buffer (final concentration 12 mM)
- 4 g NaCl (final concentration 137 mM)
- 440 mL of water

$NaIO_4$ (100 mM:): stock solution in water stored at room temperature in a dark bottle

- 2.14 g of $NaIO_4$
- 100 mL of water

Quenching buffer (4 mM $Na_2S_2O_4$ in PBS pH 6.8): stored at 4 °C, maximum 1 month in a dark bottle

- 70 mg of $Na_2S_2O_4$
- 100 mL of degassed PBS pH 6.8

Washing buffer (50 mM TRIS-HCl pH 6.8, 0.05% (wt/vol) octyl-β-D-glucopyranoside, 0.5 M NaCl, 2 mM β-mercaptoethanol): stored at 4 °C, maximum month in a dark bottle

- 10 mL of 0.5 M TRIS-HCl, pH 6.8
- 50 g octyl-β-D-glucopyranoside
- 2.92 g of NaCl
- 14 µL of β-mercaptoethanol
- 90 mL of water

SDS 0.1 % (in water): stored at room temperature in a dark bottle

- 0.1 g of SDS
- 100 mL of water

SDS 0.2 % (+ 2 mM β-mercaptoethanol in PBS pH 6.8): stored at 4 °C, maximum 1 month in a dark bottle

- 0.1 g of SDS
- 7 µL of β-mercaptoethanol
- 50 mL of PBS pH 6.8

LAEMMLI: stored at room temperature in a dark bottle

- 1.25 mL of 0.5 M TRIS-HCl, pH 6.8
- 2.5 mL of glycerol
- 2 mL of 10 % (w/v) SDS
- 2 mL of 0.5 % (w/v) Bromophenol Blue
  - → Add water to a total volume of 9.5 mL of stock solution
  - → Add 50 µL of β-mercaptoethanol to 950 µL of stock solution prior to use

DCPIP (0.4 mM working solution): store at 4 °C in in a dark bottle

- 139 mg of DCPIP
- 12 mL of degassed PBS pH 6.8
- Dilute 100 times (100 µL in 9.9 mL of degassed PBS pH 6.8) to get a 0.4 mM working solution

L-ascorbic acid sodium salt (200 mM stock solution): stored at 4 °C, maximum 1 month in a dark bottle

- 475 mg of L-ascorbic acid sodium salt
- 12 mL of degassed PBS pH 6.8
- Dilute 10 times (100 µL in 900 µL of degassed PBS pH 6.8) to get a 20 mM working solution prior to use

PBS = phosphate-buffered saline
TRIS = tris(hydroxymethyl)aminomethane
SDS = sodium dodecyl sulfate
DCPIP = 2,6-dichlorophenolindophenol

II.2. DCPIP titration

The 2,6-dichlorophenolindophenol (DCPIP) indicator [23,24] is commercially available in its oxidized blue form. To observe the colour change during oxidation, a solution of its reduced colourless form ($DCPIPH_2$) is prepared by addition of sodium ascorbate. Thus, a 20 mM solution of sodium L-ascorbate was prepared freshly before use through a 10-fold dilution of its 200 mM stock solution in degassed PBS (pH 6.8). The solution of $DCPIPH_2$ (0.4 mM) was then prepared extemporaneously by mixing 10 µL of the 20 mM L-ascorbate solution with 500 µL of the solution of the 0.4 mM DCPIP solution (Scheme S1).

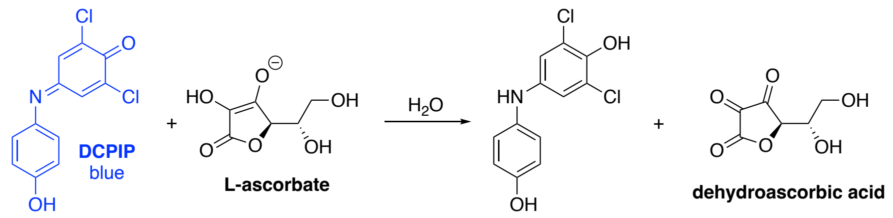

Scheme S1. Ascorbate-mediated reduction of the blue-coloured 2,6-dichlorophenolindophenol (DCPIP) into its reduced colourless form $DCPIPH_2$.

This redox indicator system can be used to check three steps of our capture protocol:

1. No colouration of the protein solution upon addition of $DCPIPH_2$ indicates that there is no excessive autoxidation.

2. Stepwise addition of $NaIO_4$ leads immediately (a few seconds) to blue colouration.

3. Addition of the quenching (reducing) $Na_2S_2O_4$ solution can be monitored by observation of decolouration.

The main aim of this titration method is the determination of the minimal amount of $NaIO_4$ required for activation of the polyphenolic probes in the presence of the respective proteome, which is thus visualized by the apparition of a blue colouration. For each proteome, a titration sample was prepared similarly to the capture samples by mixing the proteome with the polyphenolic probe (*e.g.*, **Q8**). Subsequently, the indicator was added as 100 µL of freshly prepared 0.4 mM solution of $DCPIPH_2$. Then, a stepwise addition of 10 µL (by 2 µL upon starting of colouration) of the 100 mM $NaIO_4$ stock solution was repeated until the observation of a stable blue colouration.

For capture assays, the amount of periodate used corresponds to the titration amount leading to the observation of a stable blue colouration (*i.e.*, no colour intensification upon addition of more $NaIO_4$, see Figure S1).

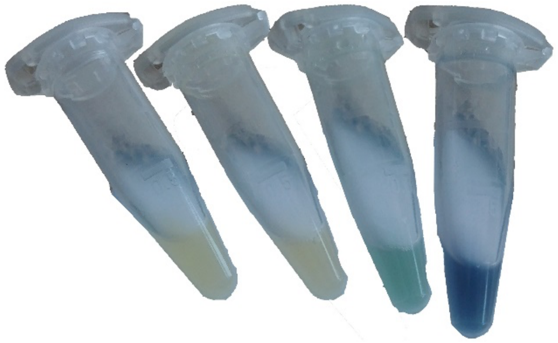

Figure S1. $DCPIPH_2$ (colourless) + $NaIO_4$ → **DCPIP** + $NaIO_3$ + H_2O (example of a qualitative titration into an Eppendorff tube).

III. Bioinformatics

Bioinformatic analyses were performed for comparing proteins identified in capture and control samples using in-house Python scripts. Only proteins marked as "Master proteins" by Proteome Discoverer were used in all of the analyses. Out of these, proteins described as "contaminant" and those identified with less than two unique peptides were discarded.

Data filtering – Abundance values were reduced by standard deviation of all measures per protein of an experiment ((*i.e.*, for all conditions included in a given experiment). After that, for each comparison:

1. For proteins whose expression was measured under both capture and control conditions, expression measure reproducibility was ensured by (i) discarding proteins with more than one missing value per condition and (ii) discarding proteins with coefficient of variation (CV) $\boldsymbol{\sigma/\mu > 0.5}$ under either of the two conditions (with $\boldsymbol{\sigma}$ the standard deviation and $\boldsymbol{\mu}$ the arithmetic mean). Remaining missing values were ignored, notably for computing $\boldsymbol{\mu}$.

2. Proteins exclusively measured under the control condition were discarded. Proteins exclusively measured under the capture condition were kept. In the rest of the manuscript, we call these proteins

"highly significant". We discarded highly significant proteins having one or more missing values under the capture condition for which they were identified to ensure the reliability of the identification.

Differential abundance – For each protein, fold change was computed as the ratio of geometrical means between capture and control triplicates of expression values. Z-scores of these ratios define the dataset denoted **F**. Differential analysis between control and capture samples was performed by first identifying the probability distribution corresponding to the best fit for **F** and then identifying the outliers. Briefly, for a list of probability distributions implemented in the Python package Scipy [36], we computed the maximum likelihood of observing **F**. Then, the best distribution fit was computed as the one minimizing the sum of square error, and p-values were computed from the cumulative distribution function of the chosen distribution with a right-tailed test and the significance threshold was set to 0.05. For proteins specific to the capture condition, the p-value was set to 0 (and fold change to 1000) to reflect that they are specifically captured by the polyphenolic probe. Proteins meeting the following requirements were considered as statistically differentially abundant: p-value less than 0.05 and no intersection between intervals of abundance measures of control and capture samples.

This process is exemplified in Figure S2 by the data obtained from interrogation of the *Helianthus* proteome database against our *Rudbeckia* protein capture results using the probe **Q8** through method **O**.

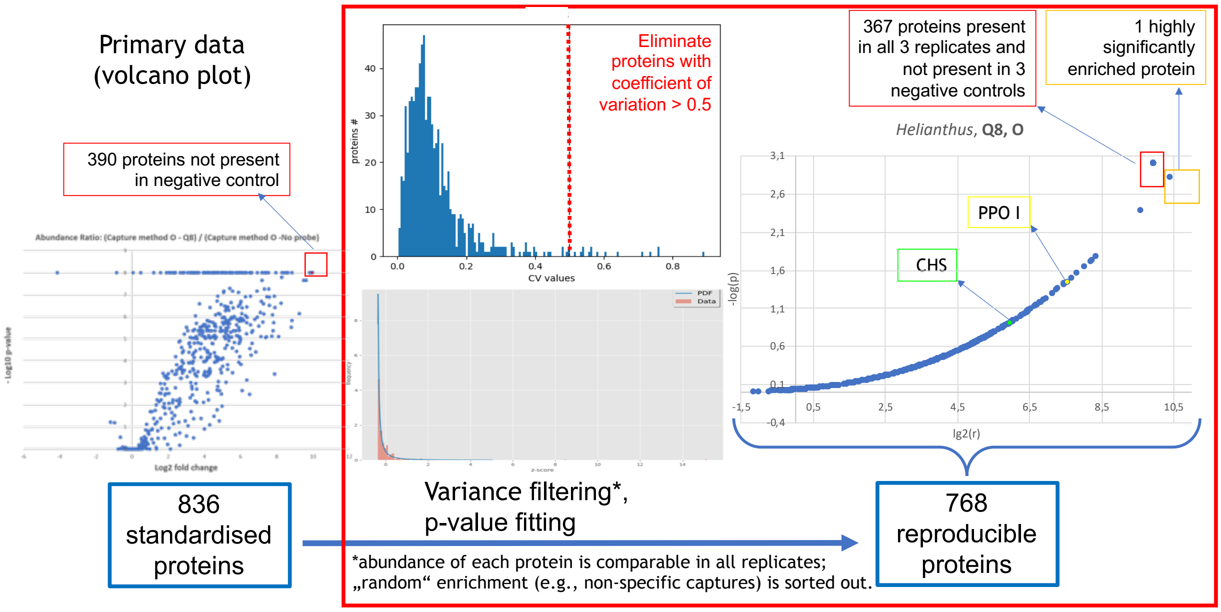

Figure S2. Bioinformatic pipeline for identification of highly significant binders of multi-target polyphenols. The main pre-cleaning step eliminates all non-reproducible proteins exceeding the threshold (0.5) of the coefficient of variation (CV) between the triplicates.

BlastP analysis - The transcriptome assembly of *Rudbeckia hirta* (assembled de novo with Trinity by the Austrian collaborating team) was used to derive the proteome. For this, Transdecoder 5.5.0 was used to identify candidate coding regions within transcript sequences. The resulting protein sequences were then used to build a species-specific database in Blastdb format (Blast v2.12.0plus) [38].

F6H search: The BlastP script (BLAST v2.12.0plus) was used to query the proteome of *Rudbeckia hirta*. The query file consisted of the F6H protein sequence from *Rudbeckia hirta* (NCBI MT875175).

F8H search: The BlastP script (BLAST v2.12.0plus) was used to query the proteome of *Rudbeckia hirta*. The query file consisted of 3 F8H protein sequences of *Scutellaria baicalensis* [https://www.ncbi.nlm.nih.gov/protein/ASW21052.1/, https://www.ncbi.nlm.nih.gov/protein/1238790556 and https://www.ncbi.nlm.nih.gov/protein/1238790554].

The following parameters were used:

```
-qcov_hsp_perc 90
-html
-evalue 1e-4
```

These parameters allow to keep the length of the protein (90% of the length of the query protein).

Table S1. Counts of all standardised, reproducible (CV < 0.5, see Figure S1), significant and highly significant proteins from *Rudbeckia hirta* after interrogation of the *Helianthus* proteome and *Rudbeckia* transcriptome databases.

Database	Counts of proteins in the capture samples				
	Method	**O**			**N**
	Probe	**Q6**	**Q8**	**K8**	**Q8**
Helianthus	Standardised	828	836	775	845
	Reproducible	754	768	404	782
	Significant	369	381	127	432
	Highly significant	355	368	121	402
Rudbeckia	Standardised	1335	1347	1214	1343
	Reproducible	1212	1239	621	1238
	Significant	492	507	180	545
	Highly significant	478	497	168	529

NB. The numbers highlighted in blue compare the captures by **Q8** and **K8** and indicate that comparable numbers of proteins were detected in the capture sample. The numbers highlighted in red indicate that the decrease of the number of proteins through the reproducibility filtering is significantly higher for **K8** than for **Q8**. We attribute this observation to the only structural difference existing between the two probes, namely the presence or absence of an oxidizable catechol group enabling the oxidative covalent addition of protein nucleophiles. In the absence of a covalent bond, the captured proteins get erratically lost during the washing. This causes a higher variation of abundance within the triplicates and thus a lower reproducibility. The fact that such a large decrease of the number of proteins is not observed when using probe **Q8** without periodate oxidation (method **N**) is attributed to the formation of covalent bonds through autoxidation or other oxidation by yet unidentified means of the highly dehydrogenation-sensitive catechol unit of the quercetin-derived moiety of **Q8**.

GO enrichment analysis – Random (non-specific) interactions of our probes with proteins would be expected to lead to the captures of proteins of different kinds with various roles and functions. A gene ontology (GO) enrichment analysis was also performed on the results of an exemplary capture experiment, namely all proteins significantly captured by probe **Q8** from *Rudbeckia* microsomes after interrogation of the *Helianthus* proteome database, in order to further qualify our capture results. This GO analysis revealed a highly significant enrichment of proteins with the general function of "catalytic activity", including catalytic functions mainly related to lyase activity and, most importantly, to oxidoreductase activity, which is in agreement with our search for flavonol hydroxylases.

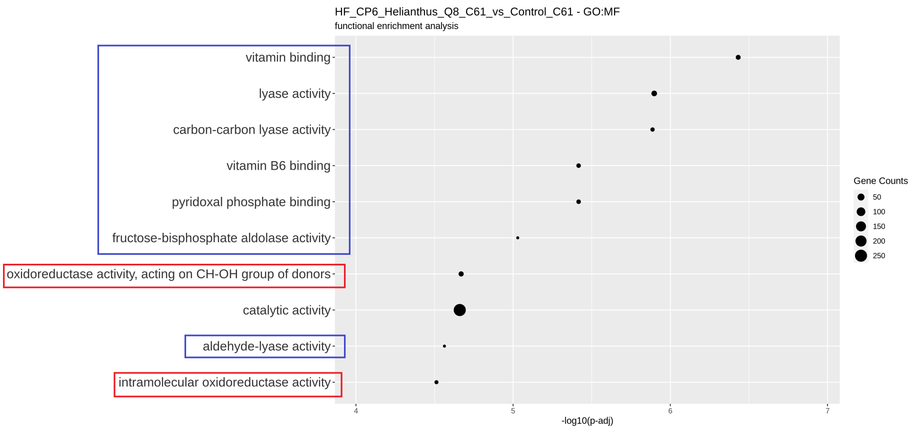

Figure S3: GO "molecular function" enrichment analysis of the proteins significantly captured by probe **Q8** from *Rudbeckia* microsomes after interrogation against *Helianthus* proteome database.

IV. NMR Spectra

Compound 4a

^{1}H / $CDCl_3$ / 300 MHz

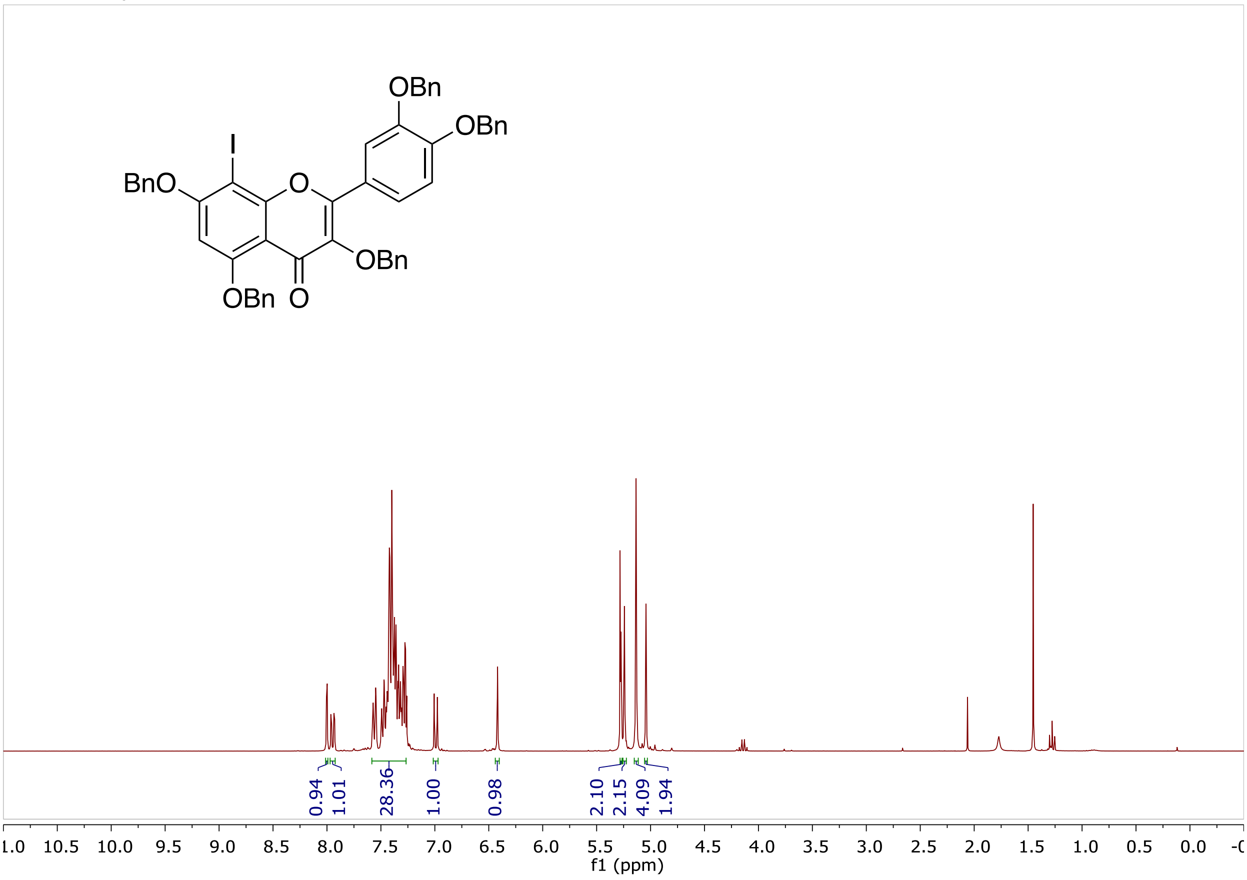

ppm	Hz	Intensity
8.00	2403.2	1139.2
8.00	2401.1	1415.1
7.96	2391.0	776.4
7.96	2388.9	577.7
7.94	2382.4	800.8
7.93	2380.3	667.9
7.62	2288.0	77.1
7.57	2273.3	1014.4
7.55	2266.2	1332.1
7.50	2251.2	668.4
7.49	2249.6	894.9
7.47	2242.9	1500.7
7.47	2241.8	1398.7
7.46	2239.4	515.4
7.45	2237.0	1076.3
7.44	2234.3	1256.4
7.44	2233.4	1190.6
7.42	2228.2	4273.4
7.42	2226.8	4067.9
7.40	2221.5	5493.2
7.38	2216.3	1908.7
7.37	2213.9	2815.1

ppm	Hz	Intensity
7.36	2210.6	2398.8
7.36	2209.7	2659.7
7.35	2208.0	1422.4
7.34	2204.0	1664.4
7.34	2202.3	1815.4
7.33	2199.5	1125.9
7.32	2197.4	1463.9
7.31	2195.3	874.3
7.31	2194.2	916.4
7.30	2190.3	1784.0
7.29	2188.0	1374.9
7.28	2184.9	2283.6
7.27	2183.0	2152.1
7.26	2179.6	1172.7
7.24	2174.3	157.4
7.23	2171.3	138.5
7.01	2103.4	1207.6
6.98	2094.7	1136.6
6.42	1927.1	1770.8
5.28	1586.2	4231.3
5.27	1583.6	2506.6
5.24	1574.0	3046.2

ppm	Hz	Intensity
5.23	1568.8	222.9
5.20	1562.5	85.1
5.13	1541.5	5734.8
5.09	1529.2	76.1
5.08	1524.5	159.9
5.04	1514.0	3094.1
5.00	1502.1	97.6
4.96	1489.2	130.9
4.18	1254.2	80.2
4.15	1247.1	241.9
4.13	1239.9	246.7
4.11	1232.8	83.2
2.06	619.0	1136.3
1.77	532.1	306.1
1.45	436.2	5198.9
1.30	390.7	339.7
1.29	386.9	105.9
1.28	383.6	634.1
1.25	376.4	309.1
0.12	35.7	82.0

Compound 4b

^{1}H / CDCl$_3$ / 300 MHz

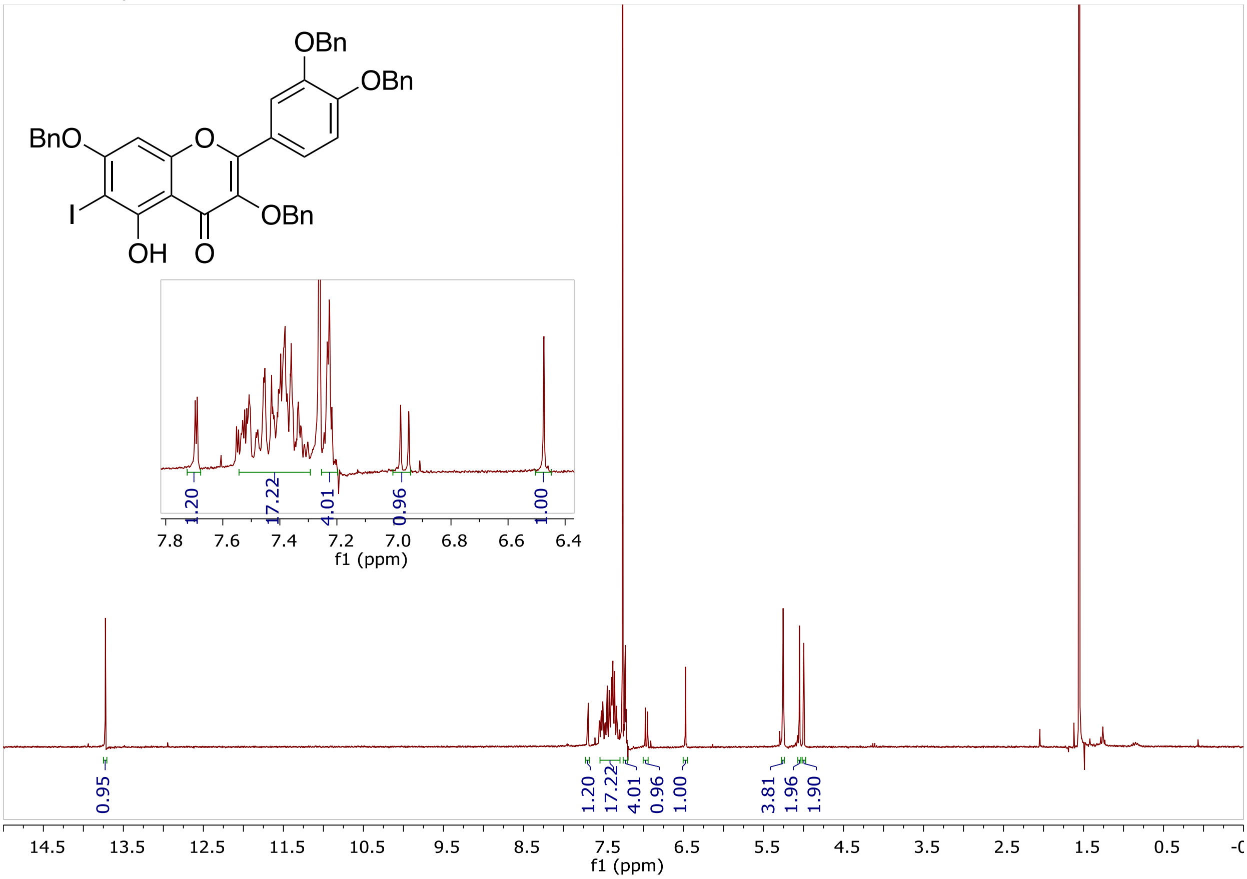

ppm	Hz	Intensity
13.94	4183.0	7.3
13.72	4119.2	239.6
12.95	3885.5	9.5
7.70	2310.0	77.3
7.69	2307.9	81.2
7.61	2282.9	18.2
7.55	2266.5	49.3
7.54	2264.4	45.7
7.53	2260.1	55.8
7.52	2257.9	67.5
7.52	2255.7	68.7
7.51	2253.4	83.7
7.48	2246.0	43.0
7.48	2244.1	45.8
7.45	2236.7	112.1
7.43	2229.6	104.5
7.43	2228.5	70.5
7.40	2220.0	127.8
7.38	2215.5	157.6
7.37	2213.3	84.6
7.36	2209.0	138.9

ppm	Hz	Intensity
7.22	2166.2	70.2
7.21	2162.6	13.9
7.19	2159.1	-23.8
6.98	2094.0	72.7
6.95	2085.3	65.9
6.91	2073.7	12.3
6.47	1943.1	146.5
6.13	1840.8	6.6
5.30	1590.7	30.1
5.25	1577.1	253.1
5.10	1531.3	8.6
5.07	1523.2	22.2
5.05	1515.6	221.4
5.00	1499.7	190.2
4.13	1240.6	7.8
4.11	1233.5	7.9
2.05	614.1	33.4
1.69	506.5	-8.2
1.62	486.5	45.0
1.55	466.5	4947.1
1.49	446.6	-41.0

7.34	2201.5	75.7
7.33	2199.0	50.2
7.31	2195.0	30.0
7.30	2191.4	32.5
7.26	2179.1	2470.4
7.24	2174.4	42.8
7.23	2171.1	140.6
7.23	2168.9	186.5

1.48	444.7	9.2
1.43	427.9	15.2
1.42	426.5	16.4
1.28	385.2	19.6
1.26	378.0	37.8
1.24	370.8	14.0
0.85	255.5	9.8
0.07	20.6	14.8

Compound 4c

^{1}H / $CDCl_3$ / 300 MHz

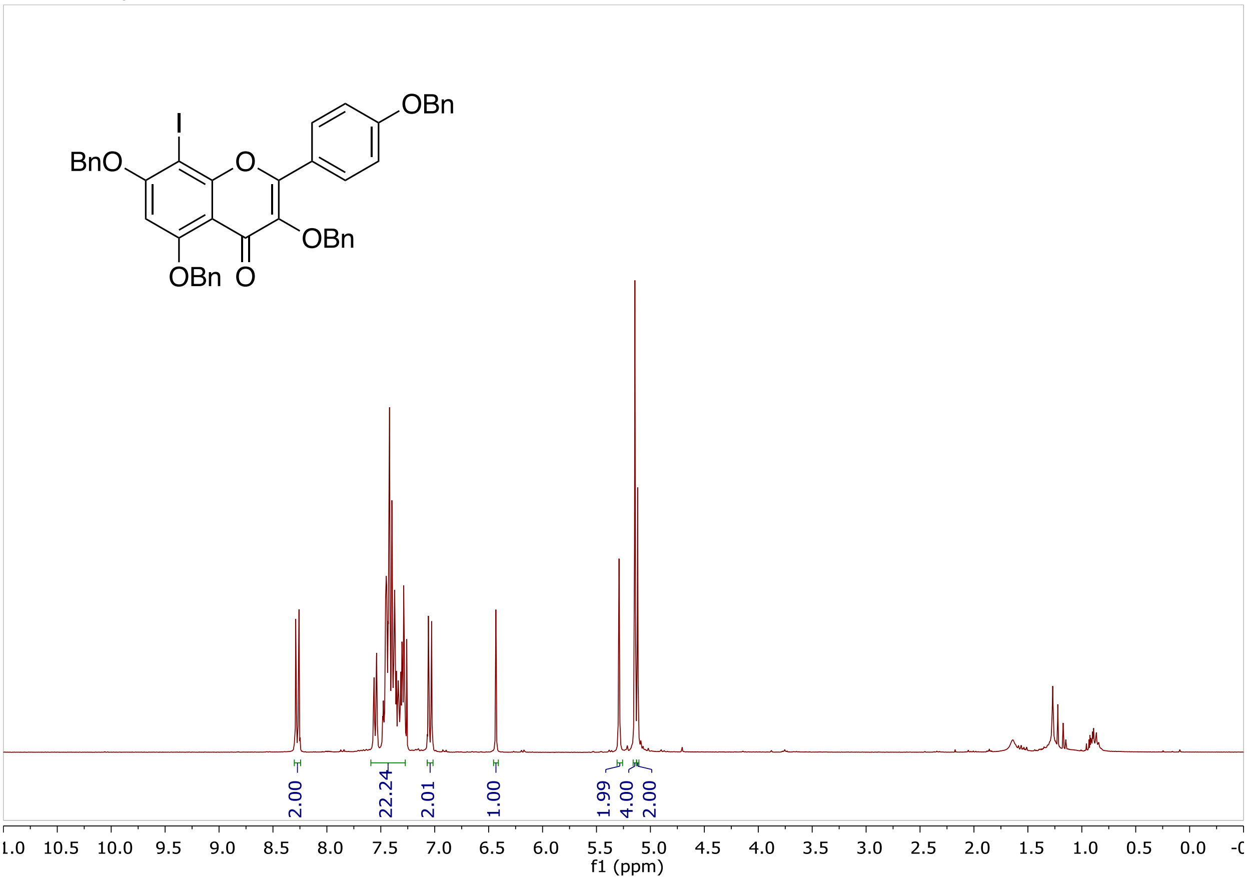

ppm	Hz	Intensity
8.30	2491.5	466.1
8.29	2488.6	3709.9
8.28	2486.6	1141.8
8.27	2481.6	1219.2
8.26	2479.6	3982.9
8.25	2476.6	414.3
7.56	2270.6	2083.8
7.54	2263.5	2765.8
7.48	2246.6	1006.4
7.48	2244.6	1440.3
7.45	2238.1	4470.1
7.45	2236.7	4905.7
7.45	2235.3	4638.1
7.43	2231.3	3657.7
7.42	2227.5	9603.7

ppm	Hz	Intensity
7.28	2185.9	3874.1
7.26	2179.6	3149.2
7.07	2122.1	503.6
7.06	2119.2	3801.9
7.05	2117.2	1181.1
7.04	2112.2	1153.1
7.03	2110.2	3649.3
7.02	2107.2	429.3
6.43	1931.4	3975.4
5.29	1588.5	5390.6
5.14	1544.3	13137.8
5.12	1536.7	7373.5
5.09	1527.9	329.2
1.64	492.2	346.7
1.30	389.8	313.0

7.40	2220.9	7015.8
7.38	2217.0	1943.9
7.37	2213.2	4519.9
7.36	2211.2	2374.6
7.36	2208.3	2264.1
7.34	2204.4	1995.6
7.34	2203.2	1995.5
7.31	2195.9	2242.9
7.30	2192.9	3080.8
7.30	2191.0	2864.3
7.29	2187.9	4645.5

1.27	381.2	1845.5
1.24	373.7	322.6
1.22	367.1	1338.8
1.17	352.1	817.2
1.15	344.8	353.6
0.93	280.2	354.1
0.92	277.4	482.6
0.91	274.7	395.4
0.90	270.5	574.3
0.89	267.3	672.8
0.87	259.7	554.4

^{13}C / $CDCl_3$ / 75 MHz

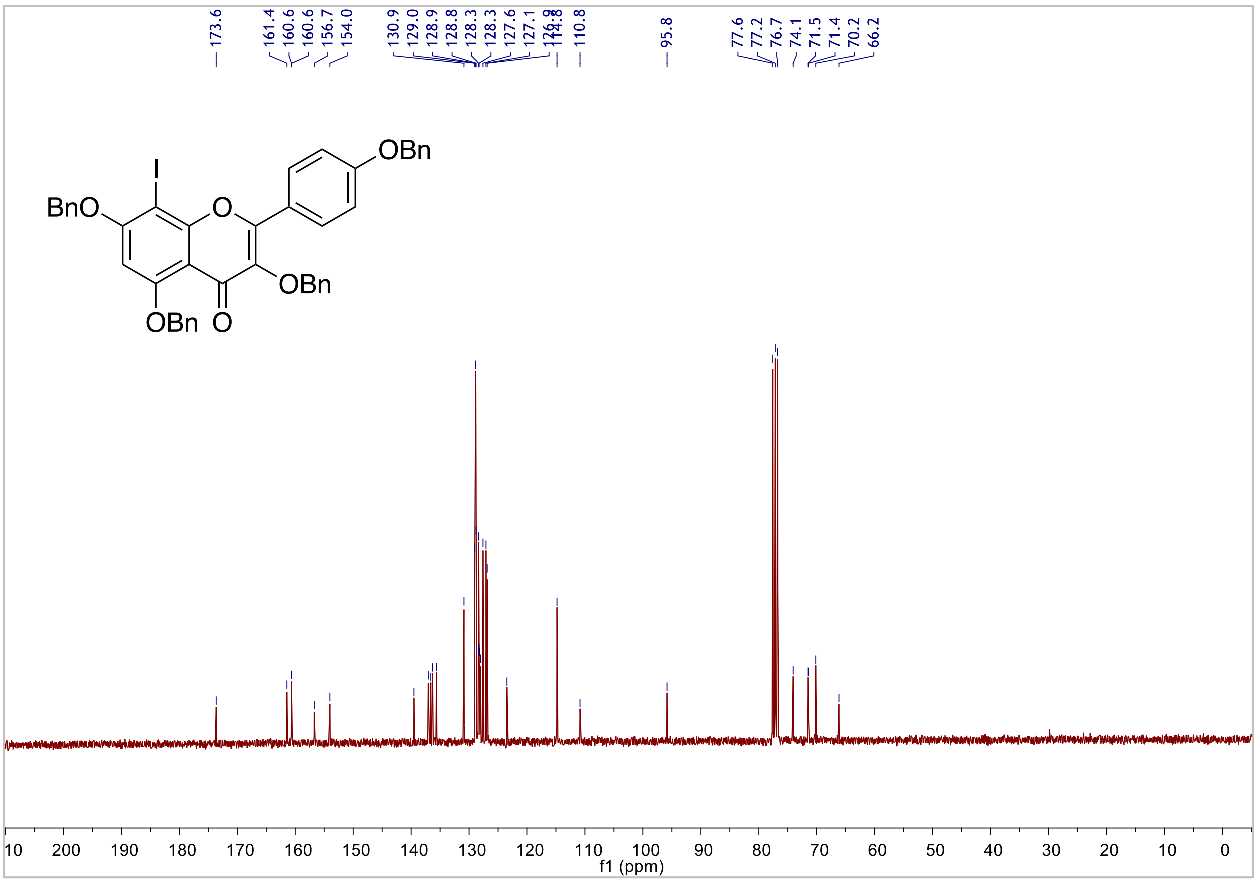

Compound 5a

^{1}H / $CDCl_3$ / 300 MHz

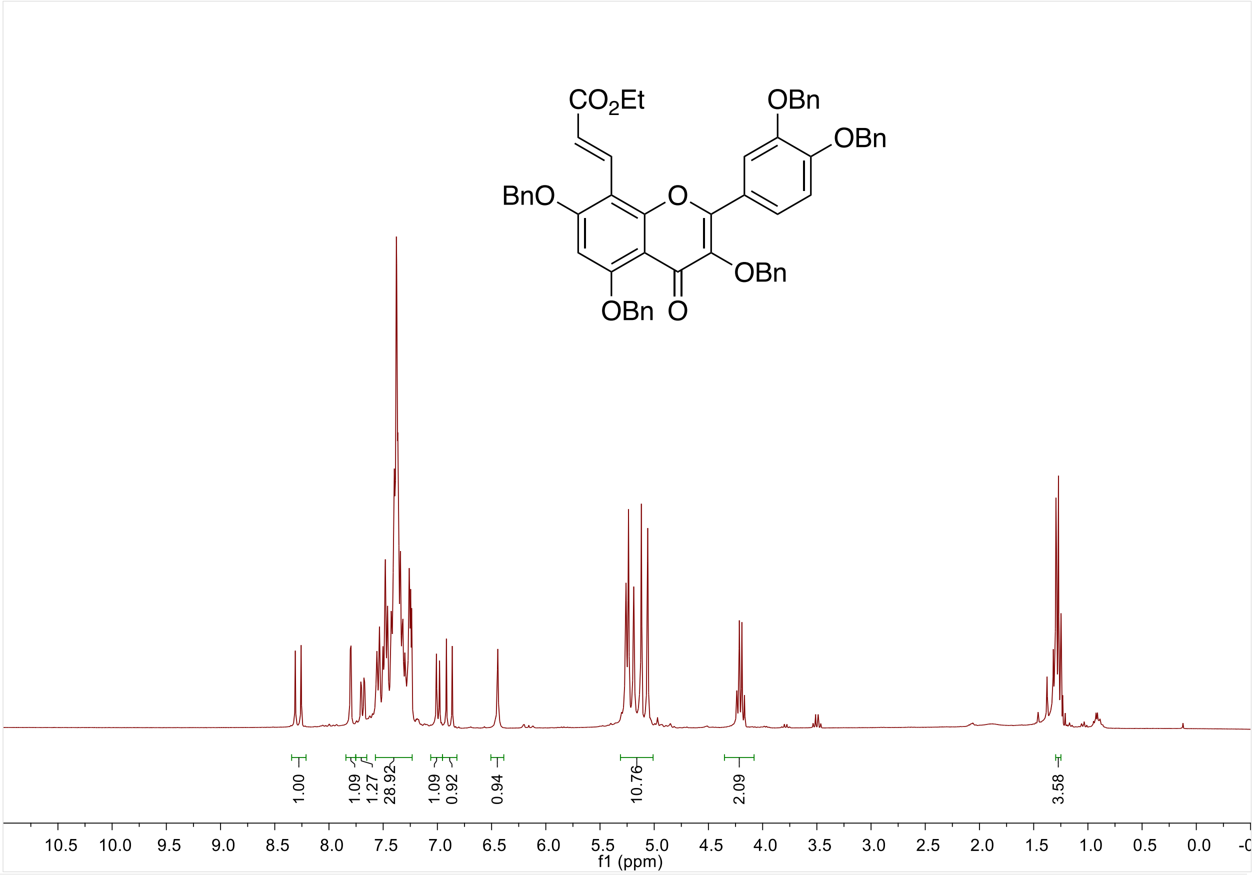

ppm	Hz	Intensity
8.31	2495.2	1311.4
8.26	2478.9	1406.9
7.80	2342.8	1337.7
7.80	2340.8	1397.9
7.71	2313.7	781.7
7.70	2311.7	694.5
7.68	2305.1	850.3
7.67	2303.1	776.9
7.62	2288.5	201.0
7.60	2281.1	240.7
7.56	2269.1	1303.9
7.53	2261.9	1723.1
7.50	2252.2	1386.1
7.48	2245.8	2870.1
7.46	2239.5	2075.9
7.43	2229.5	1988.3
7.42	2227.9	1956.8
7.40	2222.8	3827.9
7.40	2220.9	4418.9
7.38	2215.2	8379.6
7.37	2211.3	5046.8
7.35	2205.4	2563.2
7.34	2203.9	3012.4

ppm	Hz	Intensity
7.32	2198.8	1740.1
7.32	2197.2	1855.1
7.30	2191.8	1278.6
7.26	2179.6	2720.8
7.25	2175.6	2363.1
7.24	2172.8	2041.0
7.22	2169.1	298.0
7.20	2160.3	150.0
7.19	2157.8	155.8
7.18	2156.0	147.1
7.17	2153.7	141.0
7.01	2104.3	1259.5
6.98	2095.6	1144.0
6.92	2076.6	1516.7
6.86	2060.4	1392.5
6.44	1934.4	1341.0
5.30	1591.4	262.6
5.26	1579.2	2469.6
5.24	1572.4	3727.8
5.19	1557.9	2405.4
5.12	1536.7	3821.1
5.06	1519.1	3403.6
5.04	1512.2	167.2

ppm	Hz	Intensity
5.03	1509.6	130.2
4.97	1492.0	173.5
4.24	1272.5	632.0
4.21	1265.4	1830.2
4.19	1258.3	1800.3
4.17	1251.2	555.1
3.51	1054.1	224.5
3.49	1047.1	224.5
1.46	438.1	263.4
1.38	413.2	867.9
1.32	396.0	1337.9
1.29	388.6	3922.8
1.27	381.7	4304.3
1.26	376.9	739.1
1.25	374.6	1950.3
1.23	369.9	550.6
1.21	362.9	246.6
0.92	277.6	251.8
0.91	273.6	254.3
0.90	270.4	155.9
0.89	266.7	148.7

^{13}C / $CDCl_3$ / 75 MHz

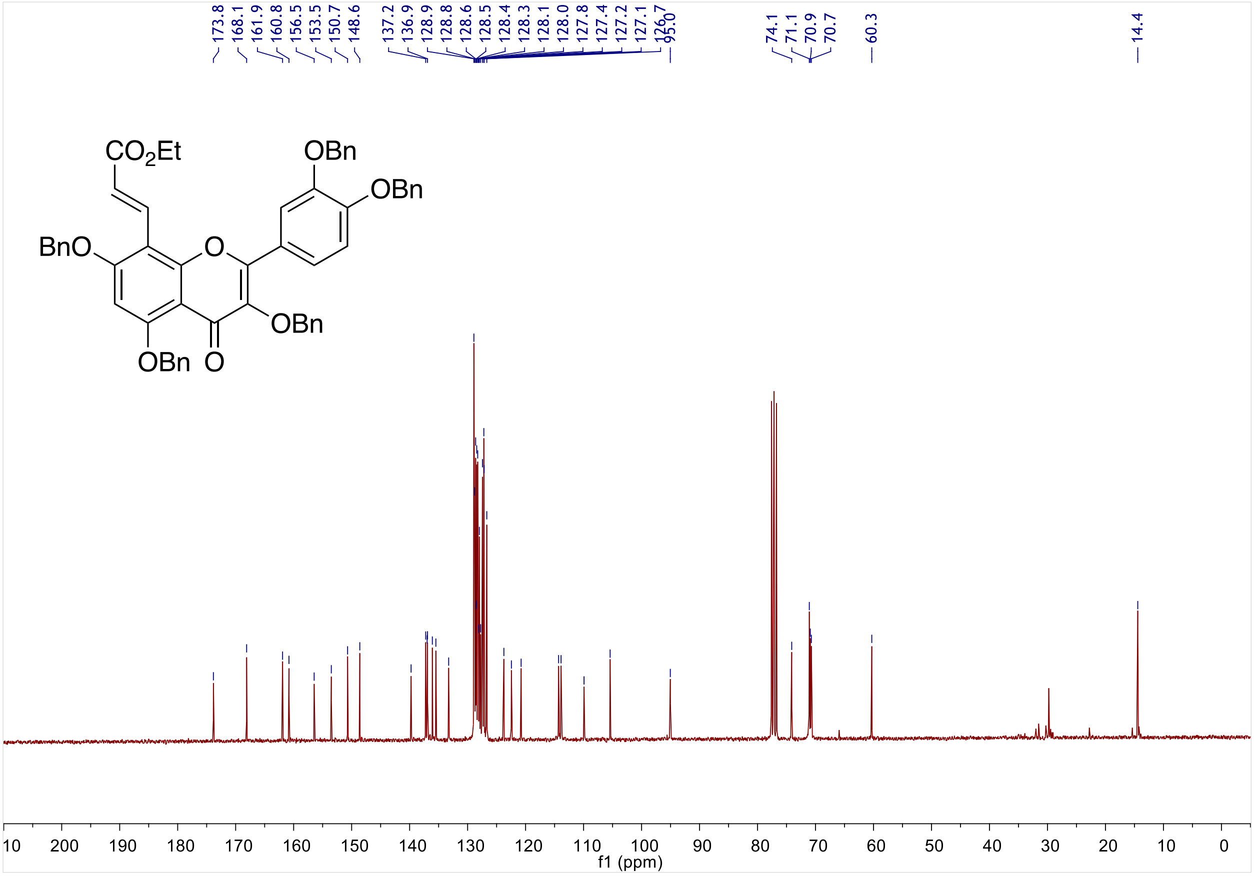

Compound 5b

^{1}H / $CDCl_3$ / 300 MHz

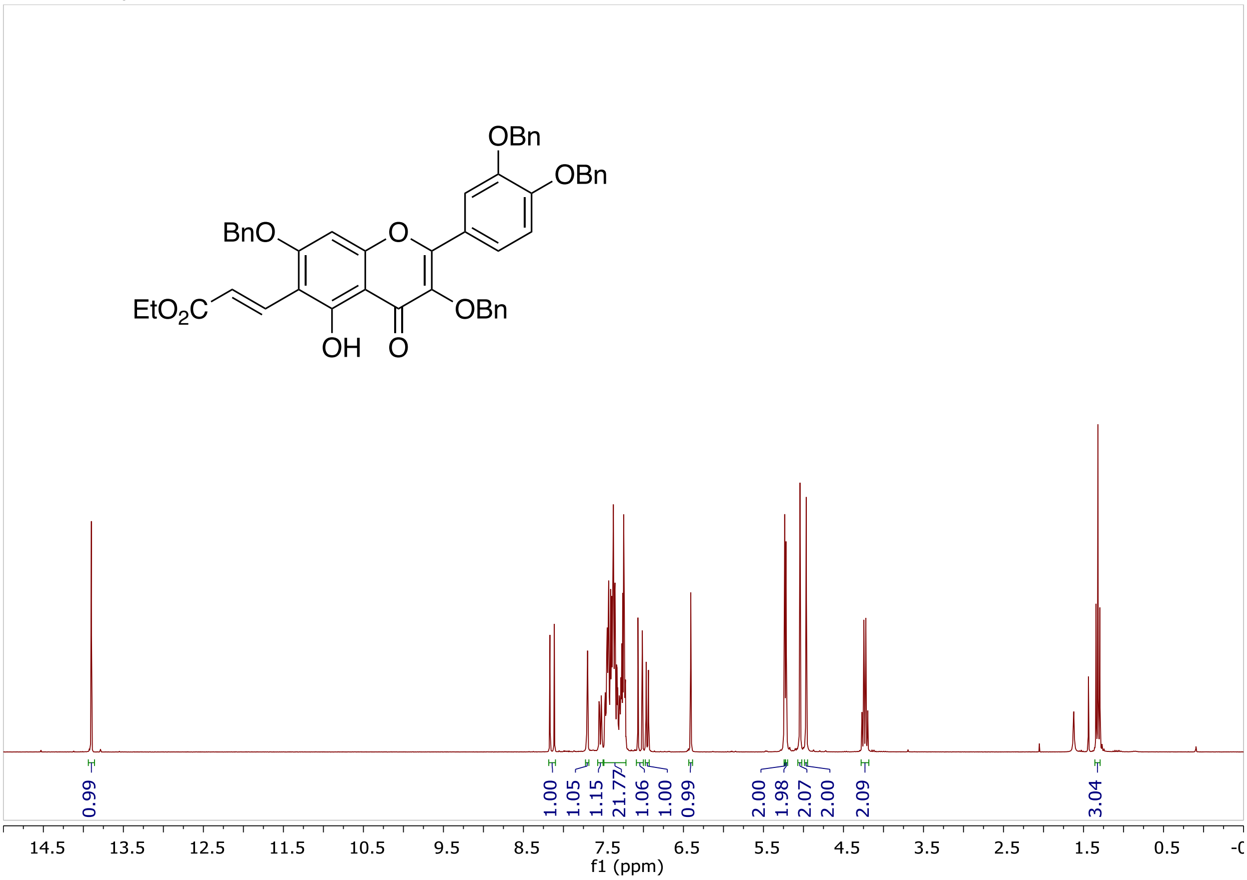

ppm	Hz	Intensity	ppm	Hz	Intensity	ppm	Hz	Intensity
13.90	4173.2	4256.5	7.36	2209.3	2976.6	6.97	2091.5	1658.2
8.17	2452.5	2156.4	7.36	2208.4	3116.0	6.94	2082.8	1507.0
8.11	2436.2	2365.6	7.35	2206.3	1645.1	6.41	1924.1	2940.0
7.71	2313.5	1749.8	7.34	2202.8	1607.6	5.23	1571.7	4377.5
7.70	2311.4	1871.2	7.33	2201.5	1267.8	5.22	1566.6	3878.7
7.56	2268.4	930.3	7.33	2200.6	1550.4	5.17	1552.7	88.6
7.55	2266.3	857.7	7.32	2198.0	1187.5	5.04	1514.1	4955.8
7.53	2259.8	1039.0	7.31	2195.5	882.3	5.00	1501.9	86.3
7.52	2257.7	987.1	7.30	2192.4	1036.4	4.97	1490.9	4696.9
7.48	2246.6	880.6	7.30	2191.5	988.2	4.27	1281.5	733.5
7.48	2244.7	1096.0	7.28	2186.5	1372.1	4.24	1274.4	2432.0
7.47	2242.4	857.5	7.27	2182.7	1997.7	4.22	1267.3	2470.7
7.46	2238.5	2261.6	7.26	2179.6	2942.6	4.20	1260.2	762.4
7.45	2237.7	2287.4	7.25	2177.9	1158.6	2.05	616.4	152.8
7.44	2233.6	2463.5	7.25	2175.9	4375.6	1.62	487.1	743.0
7.43	2232.1	3154.1	7.24	2173.1	2462.1	1.44	432.1	1388.3
7.42	2229.1	1358.7	7.23	2171.2	1125.6	1.36	408.4	105.7
7.42	2226.7	1490.6	7.22	2169.2	1335.9	1.34	403.5	2727.6
7.41	2224.9	2998.9	7.22	2167.2	286.4	1.32	396.3	6061.0
7.40	2222.5	1739.1	7.22	2166.4	266.6	1.30	389.2	2663.4
7.40	2220.6	2876.8	7.07	2122.3	2475.0	1.27	380.9	144.1
7.38	2214.6	4562.5	7.01	2105.9	2236.0	0.09	28.4	100.5

^{13}C / $CDCl_3$ / 75 MHz

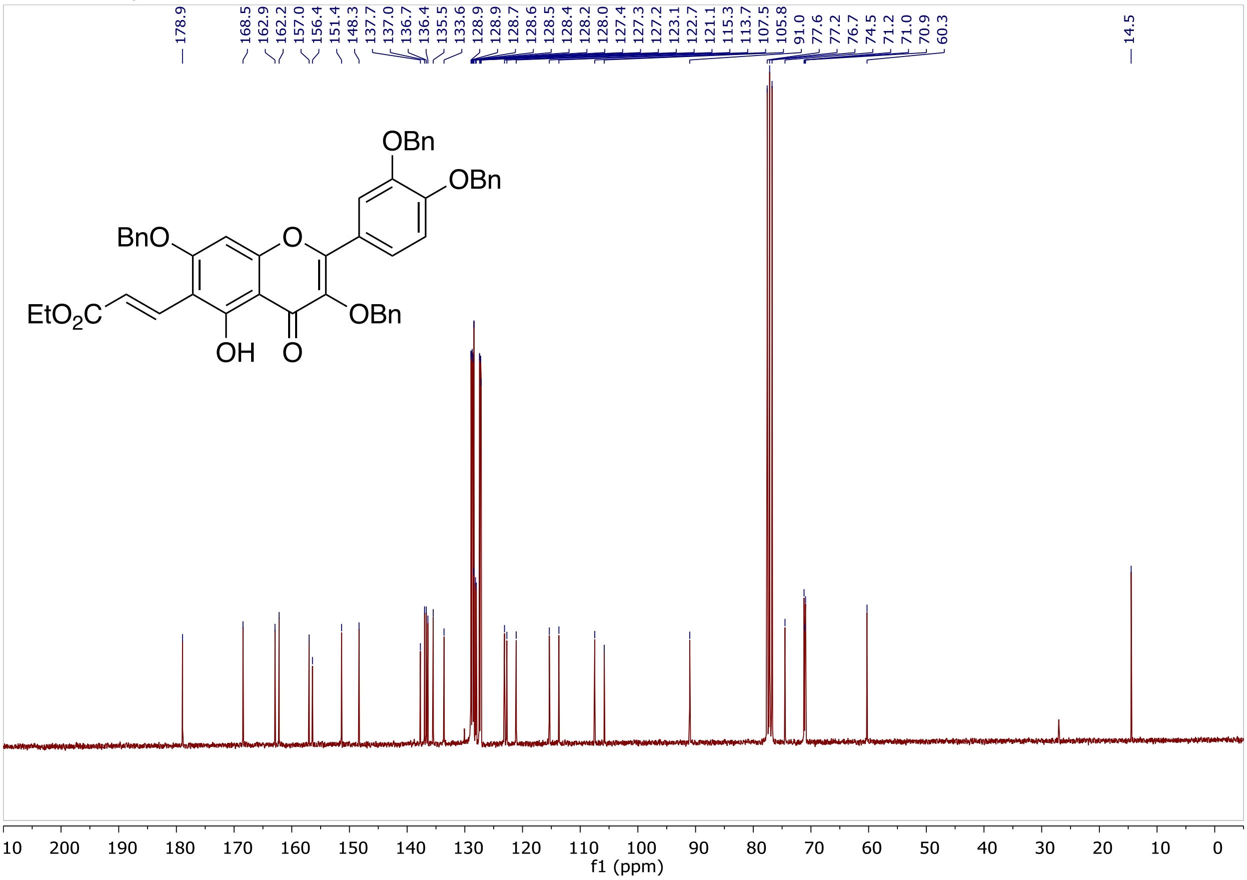

Compound 5c

^1H / $CDCl_3$ / 300 MHz

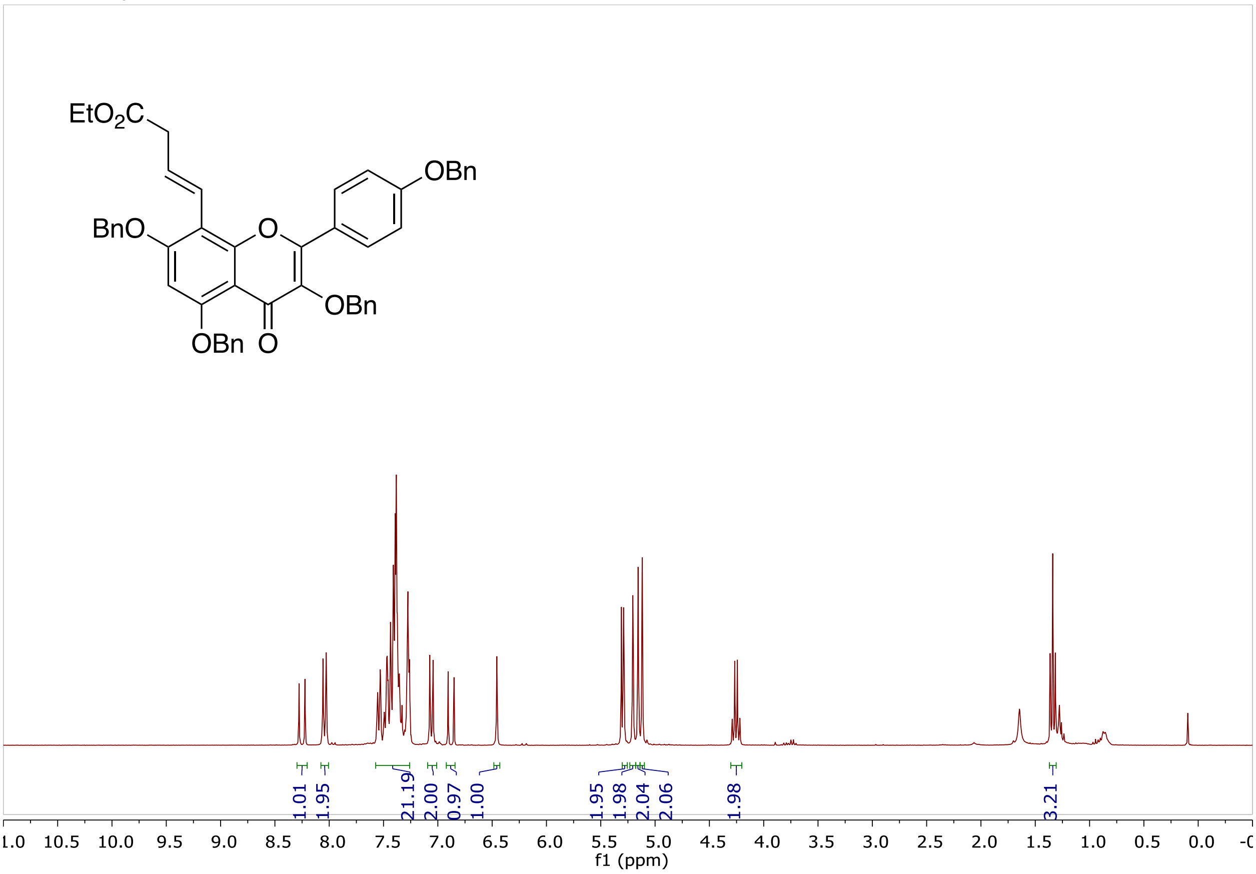

ppm	Hz	Intensity	ppm	Hz	Intensity
8.28	2484.4	128.8	6.91	2072.5	154.1
8.22	2468.1	138.2	6.85	2056.2	141.7
8.06	2418.3	180.7	6.46	1937.9	185.7
8.03	2409.3	193.6	5.31	1593.8	288.3
7.55	2267.2	110.5	5.29	1587.6	287.5
7.53	2260.0	158.0	5.20	1562.1	312.8
7.50	2250.7	49.0	5.16	1547.6	371.7
7.49	2248.8	69.4	5.12	1536.3	391.9
7.47	2242.2	182.8	5.07	1523.1	11.4
7.47	2240.9	186.5	4.29	1287.6	55.1
7.46	2238.5	136.7	4.27	1280.5	176.2
7.44	2231.7	257.0	4.24	1273.4	178.5
7.41	2223.8	376.7	4.22	1266.3	56.7
7.40	2221.0	222.5	3.75	1125.3	11.4
7.39	2218.8	483.4	3.73	1118.2	11.8
7.38	2215.6	564.3	1.65	493.8	75.9
7.37	2212.9	273.1	1.36	409.1	192.4
7.36	2208.4	148.8	1.34	401.9	399.9
7.35	2207.3	149.3	1.32	394.8	193.3
7.34	2203.2	51.5	1.28	383.4	83.6
7.33	2200.0	83.6	1.26	378.0	47.8
7.31	2192.6	32.3	1.24	371.0	24.6
7.28	2185.2	213.9	0.94	283.6	12.9
7.28	2183.6	321.2	0.92	276.5	11.5

7.27	2181.9	222.4
7.26	2178.9	179.5
7.07	2123.1	188.9
7.04	2114.1	177.9

0.90	270.4	15.5
0.88	263.0	28.8
0.86	256.6	27.2
0.10	28.7	67.3

^{13}C / $CDCl_3$ / 75 MHz

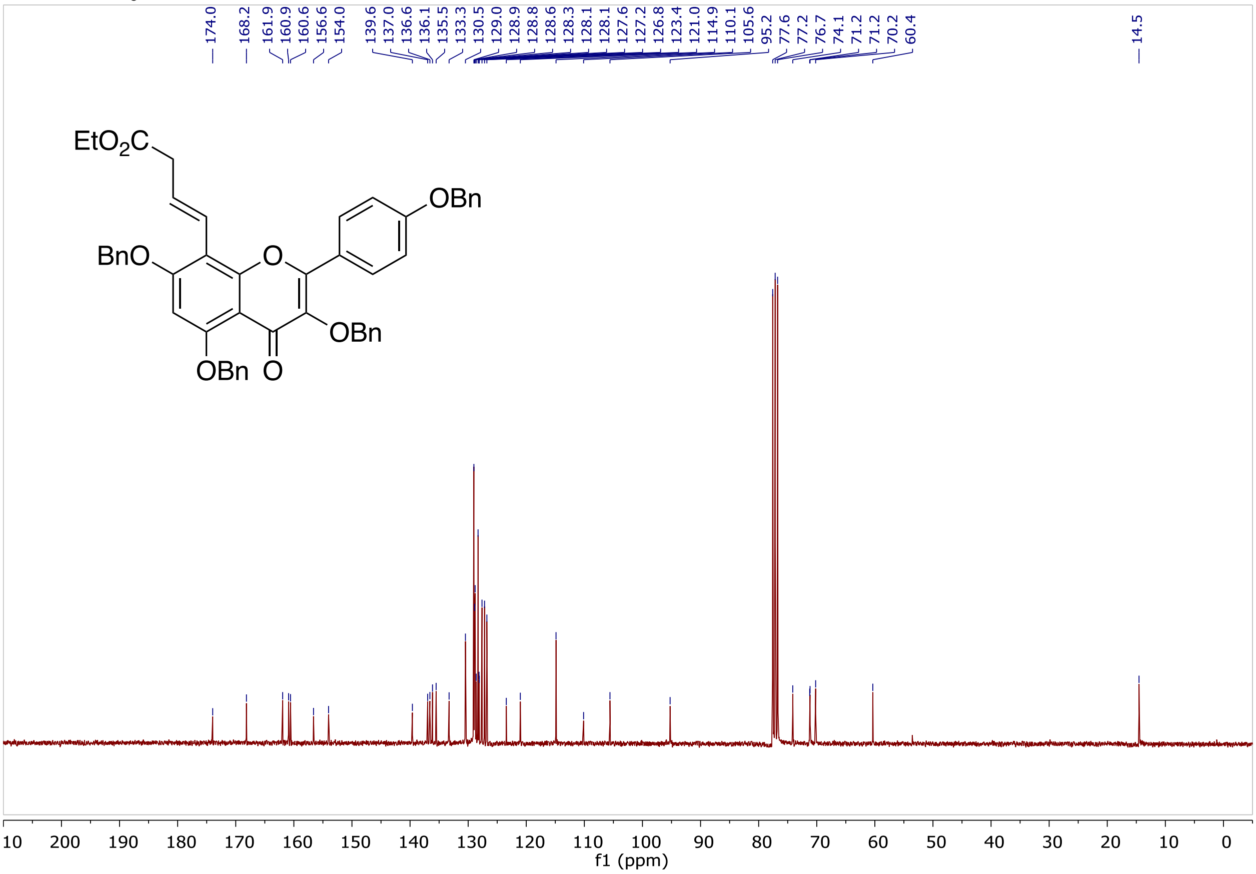

Compound 6a

^{1}H / DMSO-d_6 / 400 MHz

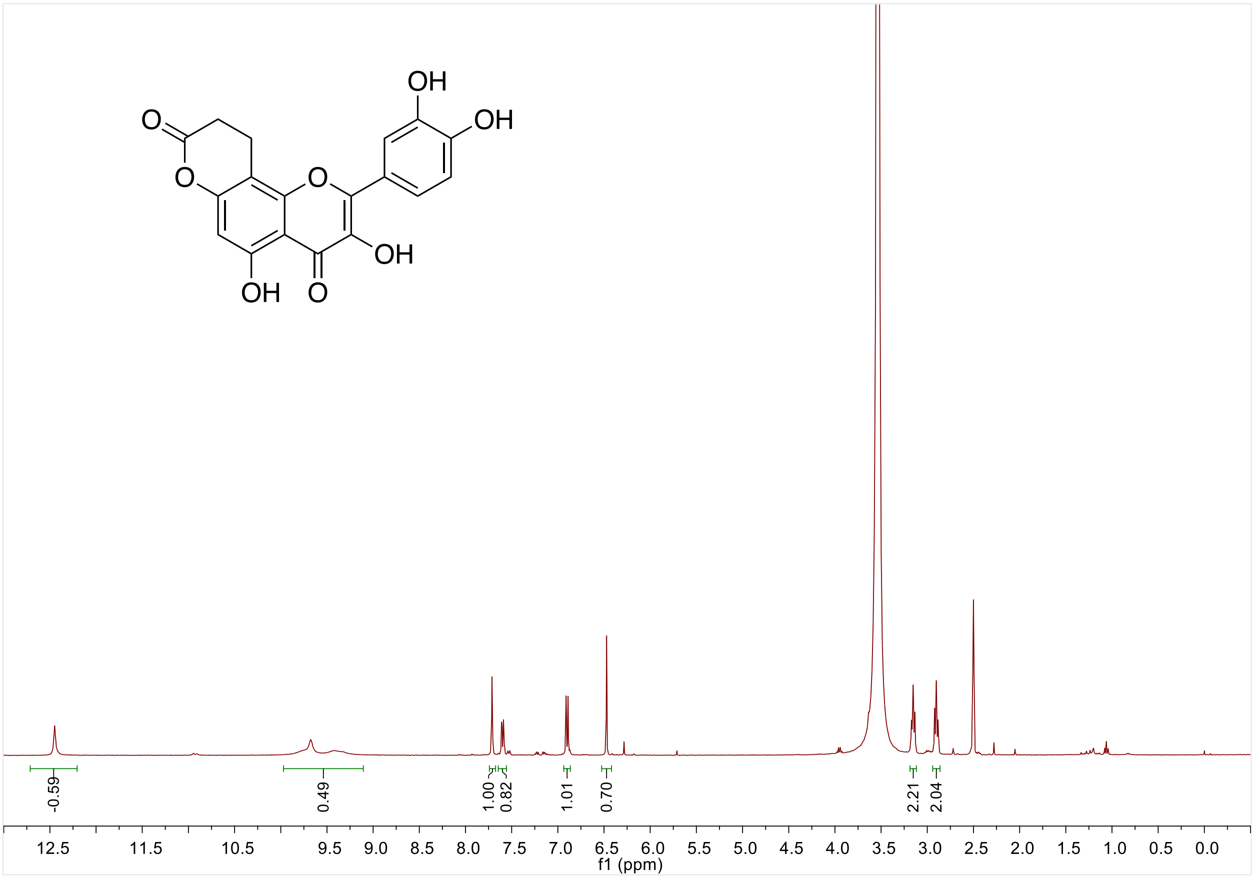

ppm	Hz	Intensity
12.45	4981.7	352.7
7.71	3086.9	999.6
7.71	3084.8	960.6
7.61	3045.4	402.8
7.60	3043.4	359.7
7.59	3037.0	432.7
7.58	3034.9	385.1
6.91	2765.6	745.1
6.89	2757.1	741.5
6.47	2590.2	1538.3
3.63	1454.1	550.3
ppm	Hz	Intensity
3.53	1414.3	137809.1
3.17	1268.9	427.6
3.16	1264.4	728.2
3.15	1261.8	889.7
3.13	1254.4	533.8
2.92	1168.7	585.3
2.90	1161.3	945.2
2.88	1154.0	430.3
2.50	1002.2	1609.4
2.50	1000.5	2009.7
2.50	998.8	1450.1

^{13}C / DMSO-d_6 / 100 MHz

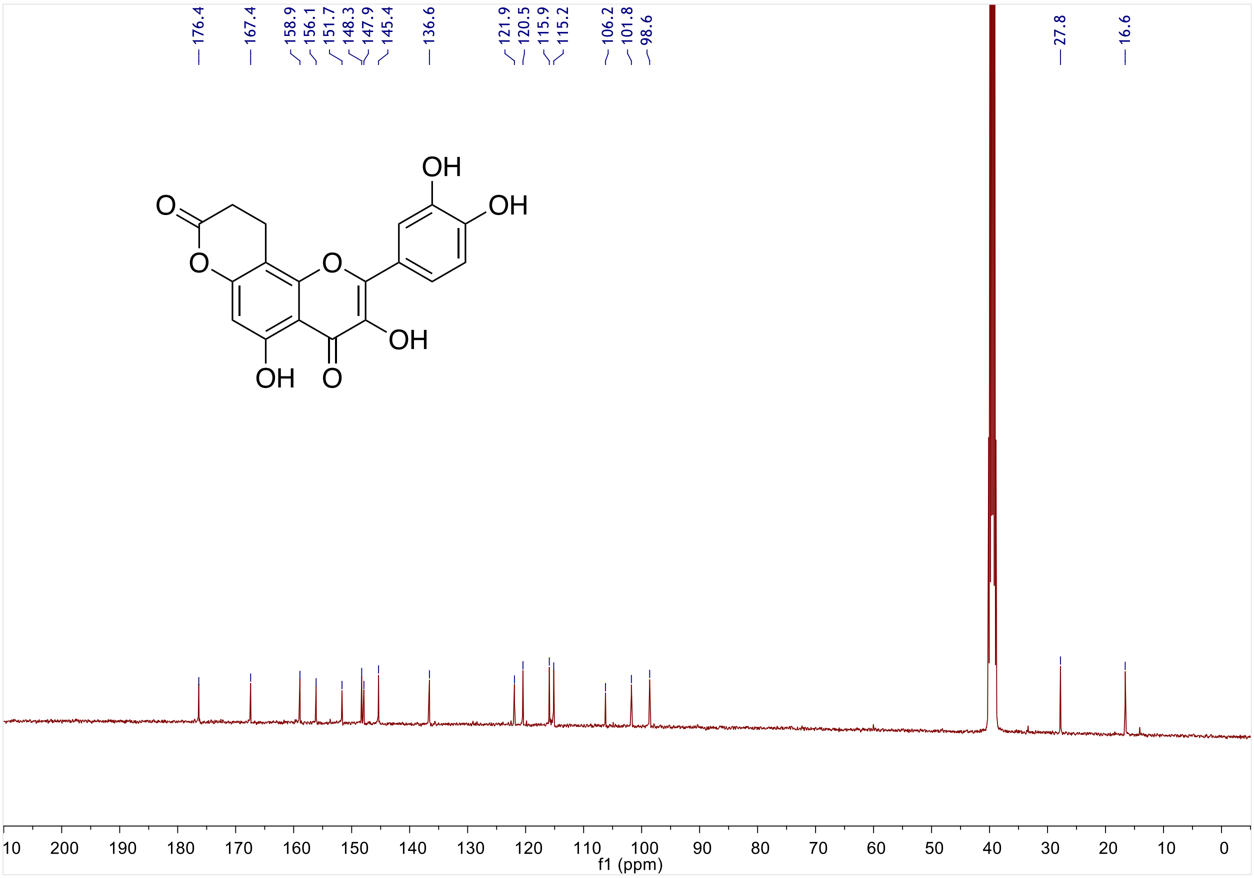

Compound 6b

^{1}H / DMSO-d_6 / 300 MHz

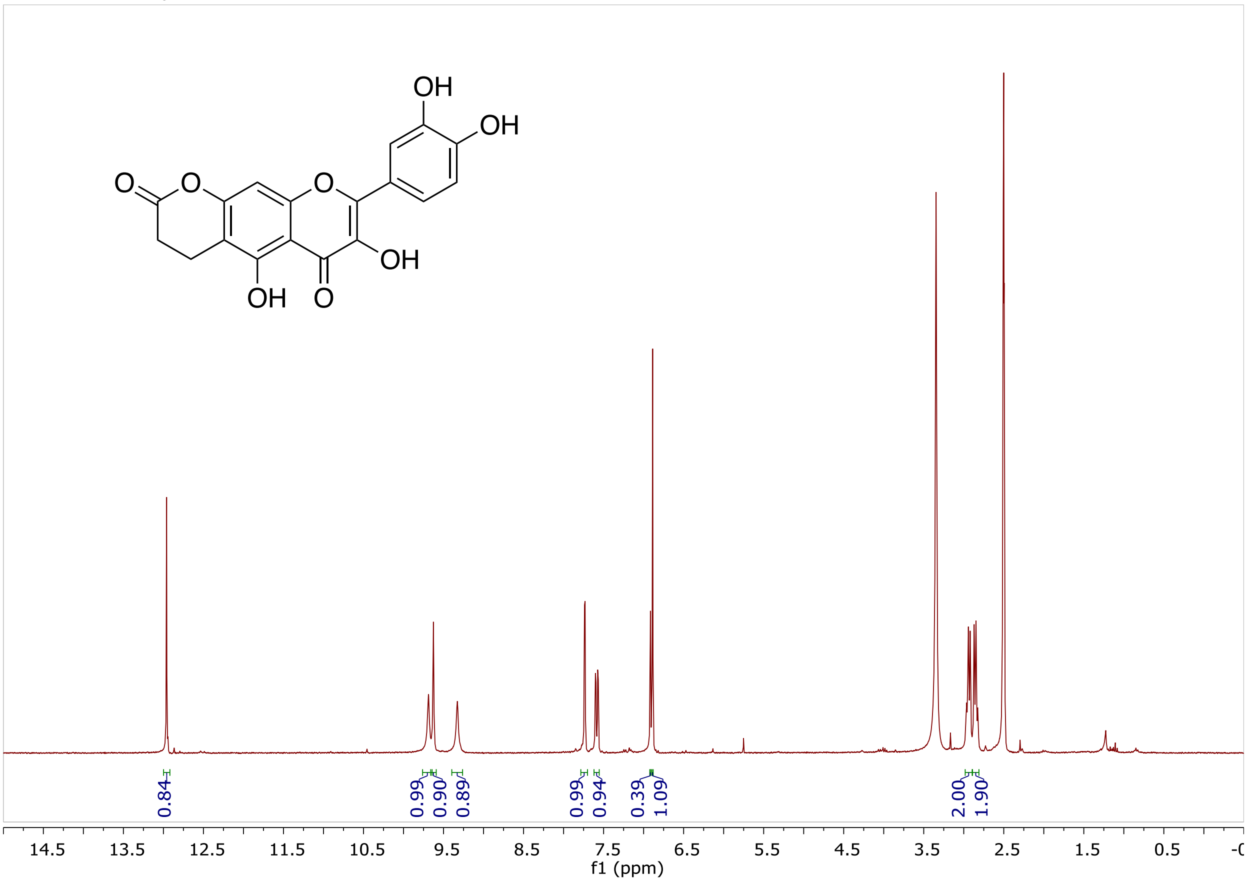

ppm	Hz	Intensity
12.96	3891.0	1860.7
9.69	2908.7	427.4
9.63	2890.2	952.7
9.33	2800.4	377.1
7.74	2323.4	1075.9
7.73	2321.2	1103.2
7.60	2282.4	580.7
7.59	2280.2	512.6
7.57	2273.9	605.4
7.57	2271.7	546.5
6.92	2076.1	1034.4
6.89	2067.6	2931.4
3.35	1004.5	4067.8
ppm	Hz	Intensity
3.17	950.7	148.3
2.96	889.6	367.2
2.94	883.5	918.1
2.92	876.6	887.3
2.87	861.7	934.2
2.85	856.1	834.4
2.85	854.7	961.5
2.83	848.5	331.7
2.82	846.5	286.9
2.51	752.4	3999.0
2.50	750.7	4937.3
2.49	749.0	3415.9
1.23	368.0	165.6

^{13}C / DMSO-d_6 / 75 MHz

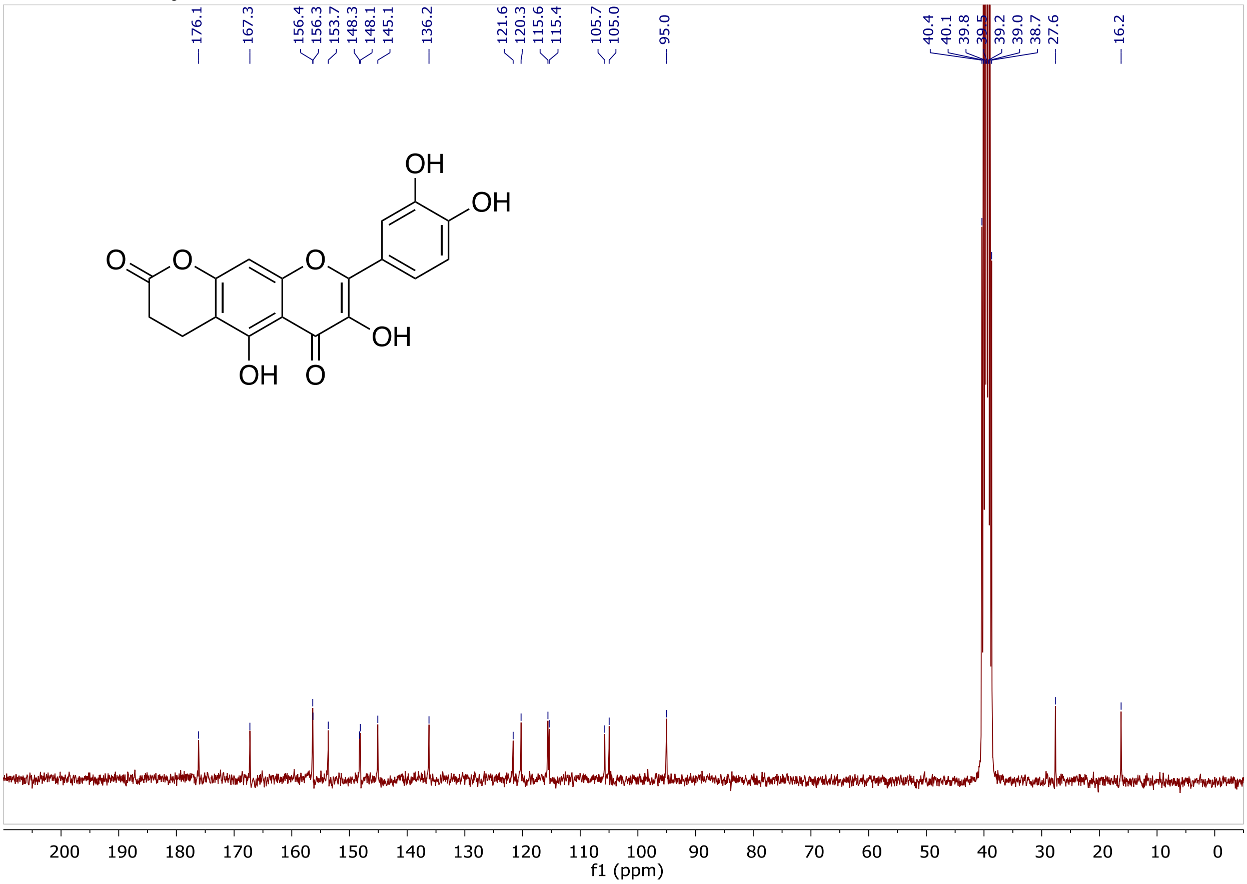

Compound 6c

^{1}H / DMSO-d_6 / 400 MHz

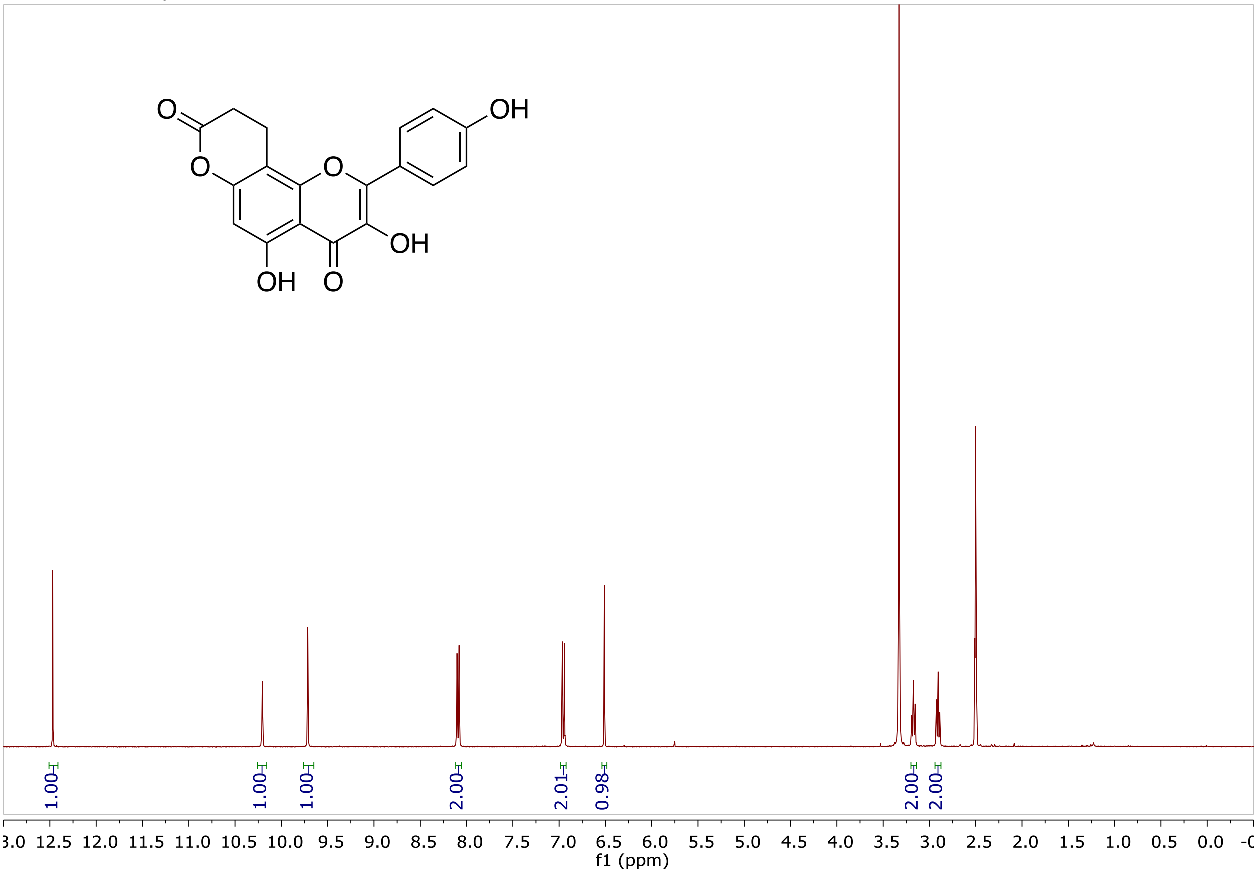

ppm	Hz	Intensity
12.47	4990.0	4486.8
10.21	4084.2	1658.7
9.71	3887.8	3035.4
8.10	3242.5	2375.3
8.08	3233.6	2583.8
6.97	2787.7	2679.9
6.94	2778.7	2638.8
6.51	2606.0	4103.3
3.33	1331.9	20596.8
3.19	1277.1	797.0
3.17	1269.9	1682.0
3.15	1262.4	1092.2
2.92	1170.5	1198.4
2.91	1162.8	1908.6
2.89	1155.7	880.8
2.52	1007.8	274.4
2.51	1004.3	2759.0
2.50	1002.4	5910.5
2.50	1000.6	8152.7
2.50	998.8	5789.7
2.49	996.9	2645.9

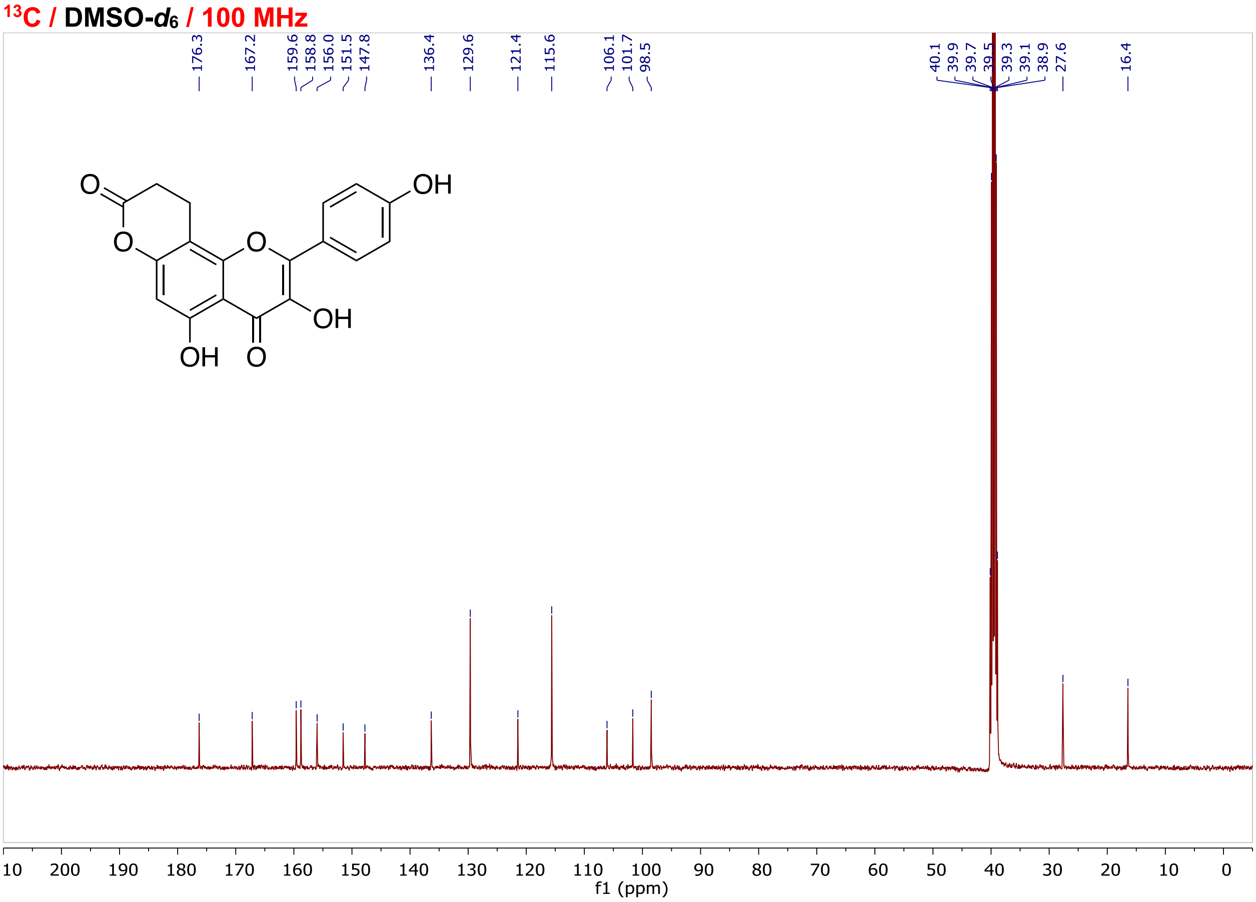

Probe Q8

^{1}H / Methanol-d_4 / 600 MHz

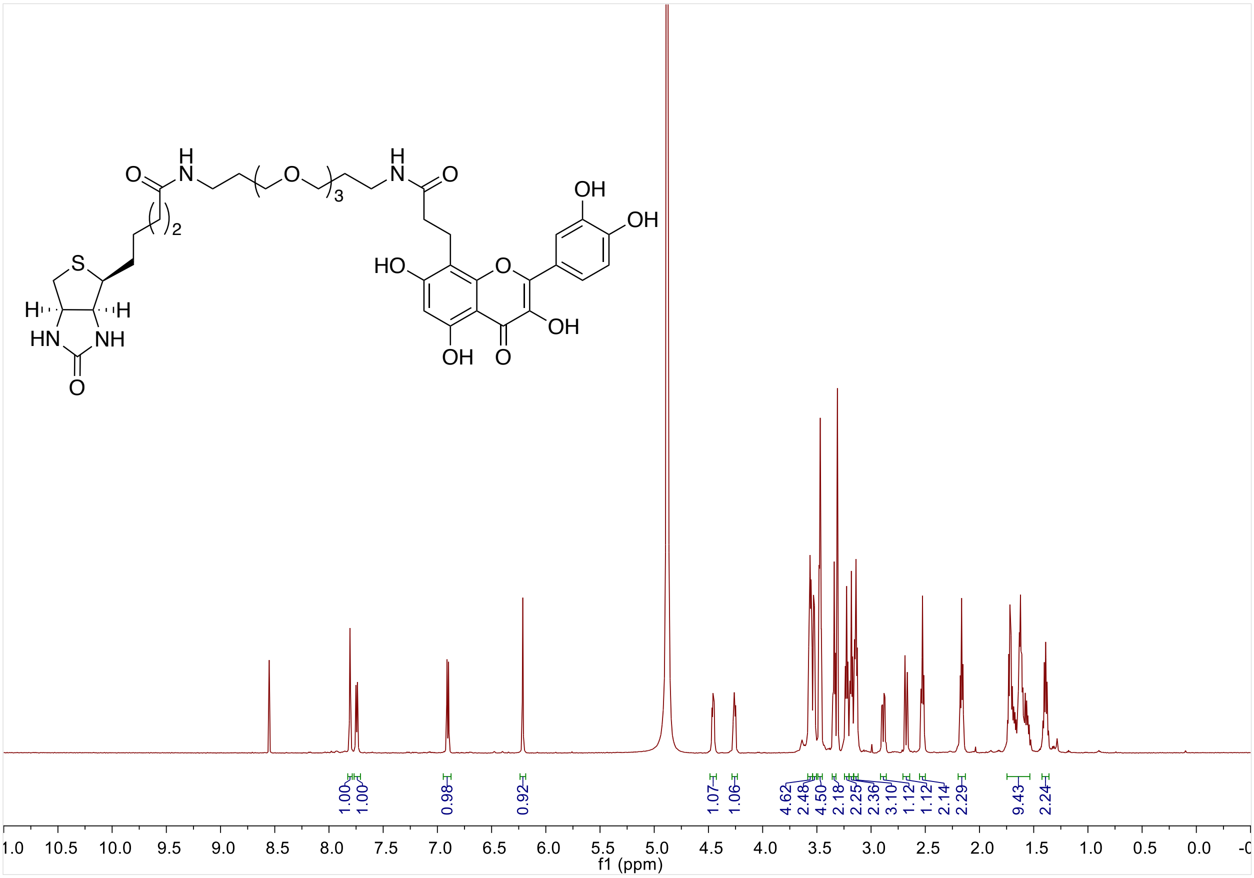

ppm	Hz	Intensity	ppm	Hz	Intensity	ppm	Hz	Intensity
8.55	5132.2	454.2	3.47	2081.8	1646.2	2.18	1306.5	376.4
7.81	4684.9	610.2	3.35	2010.6	484.2	2.16	1299.3	757.7
7.75	4652.2	328.9	3.34	2004.6	937.8	2.15	1291.9	432.0
7.74	4643.8	343.9	3.33	1998.5	488.0	1.74	1044.8	156.7
6.91	4147.8	455.7	3.31	1986.8	1792.6	1.73	1038.4	482.3
6.90	4139.3	443.7	3.24	1942.5	425.7	1.72	1031.9	726.5
6.21	3728.4	759.8	3.23	1935.8	816.6	1.71	1025.4	573.8
4.88	2928.9	14410.6	3.21	1929.0	444.1	1.70	1018.8	323.3
4.47	2681.1	217.0	3.19	1916.1	474.4	1.69	1011.3	223.3
4.46	2675.6	289.1	3.18	1909.5	892.4	1.67	1004.6	198.3
4.45	2668.7	245.0	3.17	1902.9	470.6	1.66	997.6	159.1
4.27	2562.3	260.9	3.15	1891.1	601.2	1.63	979.5	589.1
4.26	2557.8	292.8	3.14	1883.8	950.3	1.62	973.4	774.5
4.26	2554.7	252.3	3.13	1877.0	513.8	1.61	967.8	648.4
4.25	2550.2	228.7	2.90	1741.5	228.7	1.60	961.2	418.2
3.57	2143.2	800.6	2.89	1736.6	232.2	1.59	954.4	279.7
3.56	2138.4	970.4	2.88	1728.8	289.5	1.58	947.1	290.3
3.55	2132.7	849.9	2.87	1723.9	271.8	1.57	939.4	252.3
3.55	2127.9	692.6	2.69	1612.6	475.9	1.55	932.8	178.8
3.53	2119.0	773.1	2.67	1599.9	392.6	1.41	849.1	153.5
3.53	2116.9	740.9	2.54	1522.9	395.0	1.40	841.6	441.8
3.52	2114.8	771.5	2.53	1515.8	769.7	1.39	833.9	541.8
3.48	2087.5	1179.2	2.51	1508.5	377.6	1.38	826.2	344.7

^{13}C / Methanol-d_4 / 150 MHz

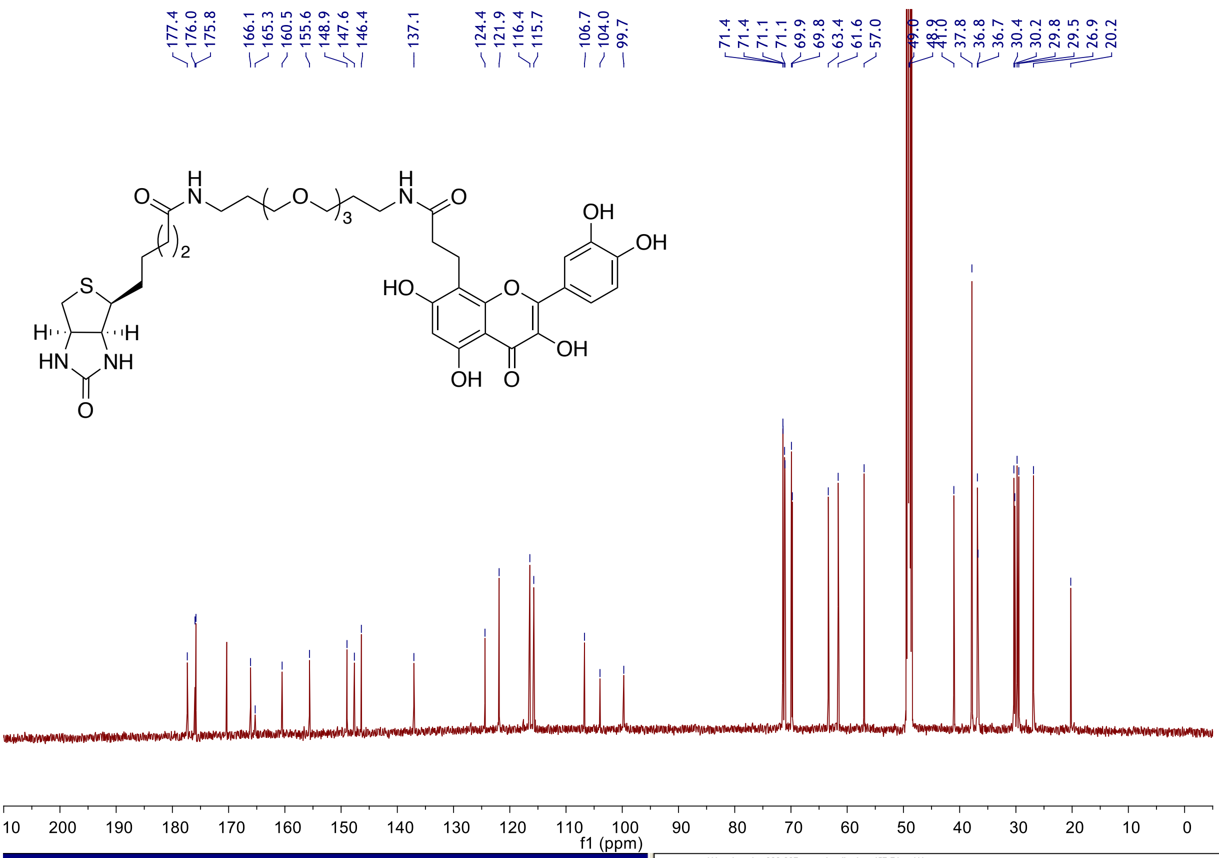

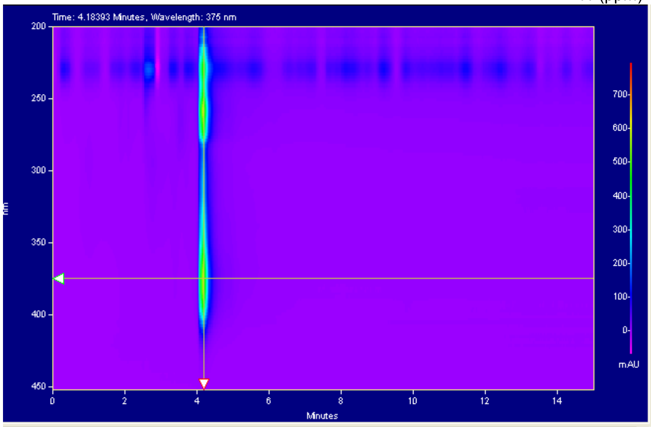

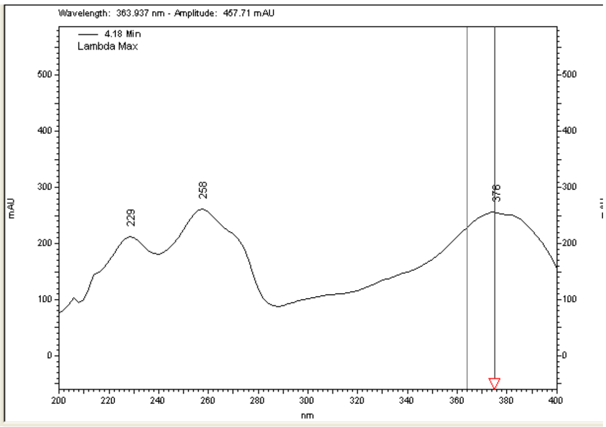

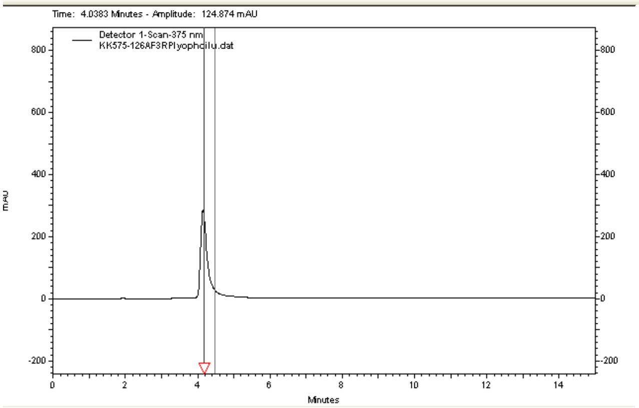

Top left: HPLC profile of UV absorption of probe **Q8**.
Bottom left: HPLC chromatogram at the local absorbance maximum of probe **Q8**.
Top right: UV absorption spectrum of probe **Q8**.

Probe Q6

^{1}H / Methanol-d_4 / 400 MHz

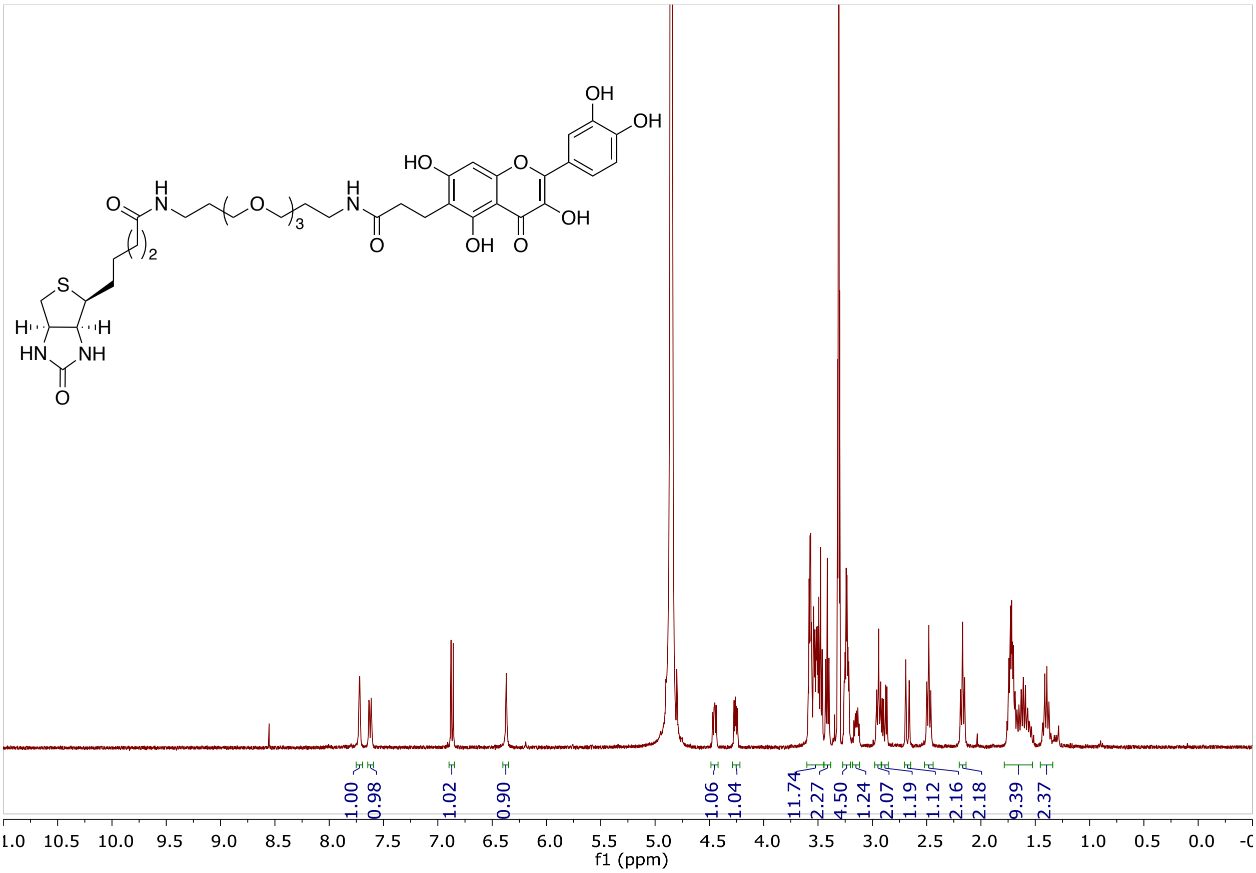

ppm	Hz	Intensity	ppm	Hz	Intensity	ppm	Hz	Intensity
8.55	3423.1	103.1	3.51	1404.4	470.0	2.69	1077.5	394.3
7.72	3089.1	318.3	3.50	1401.5	544.9	2.66	1064.8	300.0
7.63	3055.5	209.5	3.49	1397.9	677.9	2.50	1000.0	293.1
7.63	3053.6	186.2	3.48	1391.7	904.5	2.48	992.6	549.5
7.61	3047.0	219.4	3.46	1385.5	440.7	2.46	985.3	253.8
7.61	3045.1	197.2	3.43	1372.8	396.1	2.19	875.8	259.5
6.88	2752.9	483.2	3.41	1366.6	856.3	2.17	868.3	565.0
6.86	2744.4	468.1	3.40	1360.3	402.2	2.15	860.9	313.1
6.37	2549.1	331.9	3.26	1303.7	391.9	1.74	697.9	400.9
4.47	1788.7	155.6	3.25	1300.8	429.5	1.73	693.8	477.1
4.46	1783.9	175.8	3.24	1297.0	809.7	1.73	691.4	638.7
4.45	1780.7	198.7	3.23	1294.0	777.2	1.72	687.5	662.8
4.44	1776.2	187.5	3.22	1290.3	451.7	1.71	685.1	476.3
4.27	1710.0	209.8	3.22	1287.2	385.0	1.70	681.2	458.7
4.26	1705.6	224.6	3.17	1268.5	117.8	1.69	674.4	249.5
4.25	1702.2	183.1	3.16	1262.8	149.6	1.67	667.4	167.1
4.24	1697.7	173.7	3.15	1259.3	161.2	1.65	660.5	188.9
3.58	1433.5	761.4	3.13	1253.8	176.8	1.63	652.1	259.0
3.57	1430.3	954.8	3.12	1249.4	102.4	1.61	644.5	313.8
3.57	1427.5	967.1	2.96	1184.7	256.5	1.59	636.6	277.9
3.56	1425.2	563.8	2.94	1177.5	533.3	1.57	628.6	175.0
3.54	1417.6	634.9	2.92	1170.0	294.0	1.43	572.9	107.0
3.54	1415.0	464.2	2.91	1164.4	218.6	1.41	565.5	330.1
3.53	1412.0	530.7	2.90	1159.4	215.0	1.39	557.7	363.6

3.52	1408.6	496.3	2.88	1151.6	278.9	1.37	549.9	202.4
3.51	1406.7	543.1	2.87	1146.6	270.5			

^{13}C / Methanol-*d*$_4$ / 100 MHz

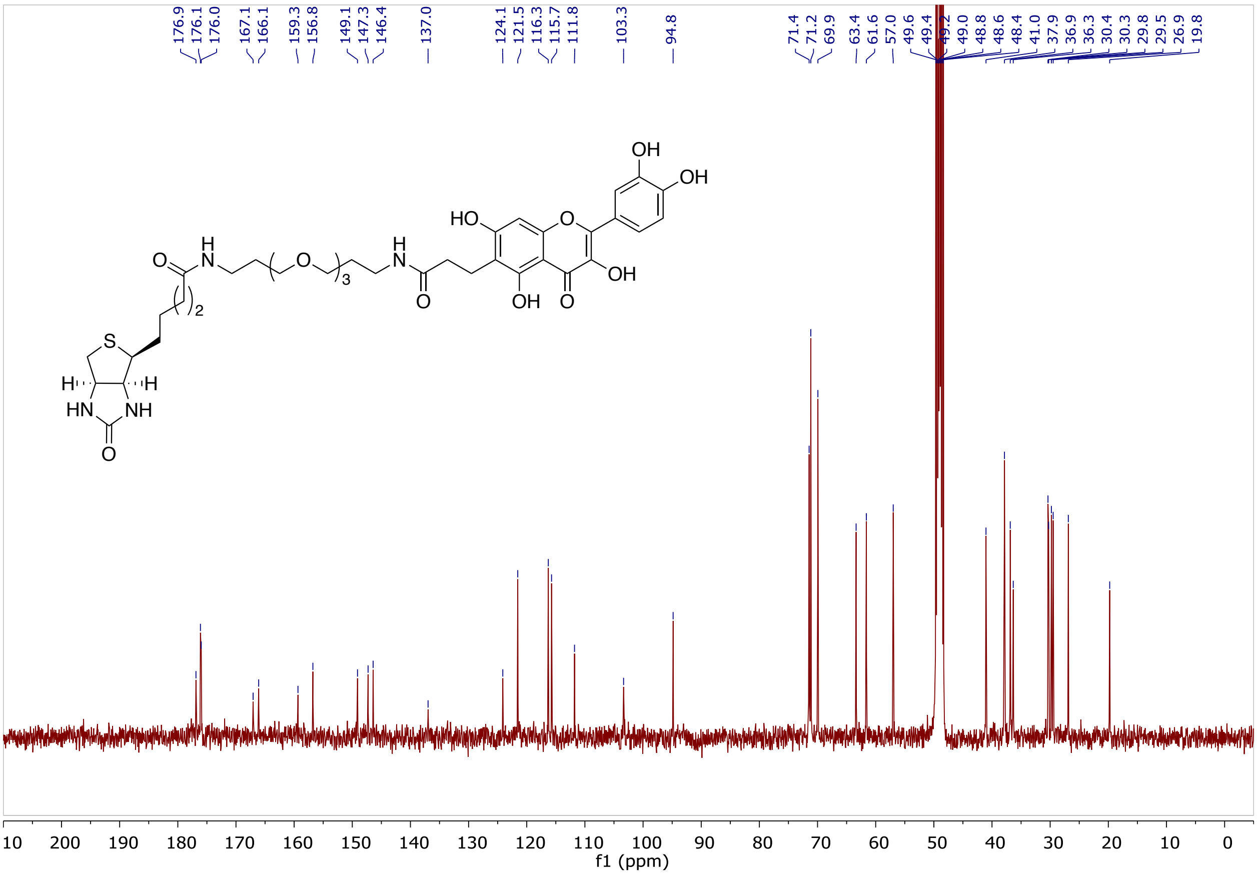

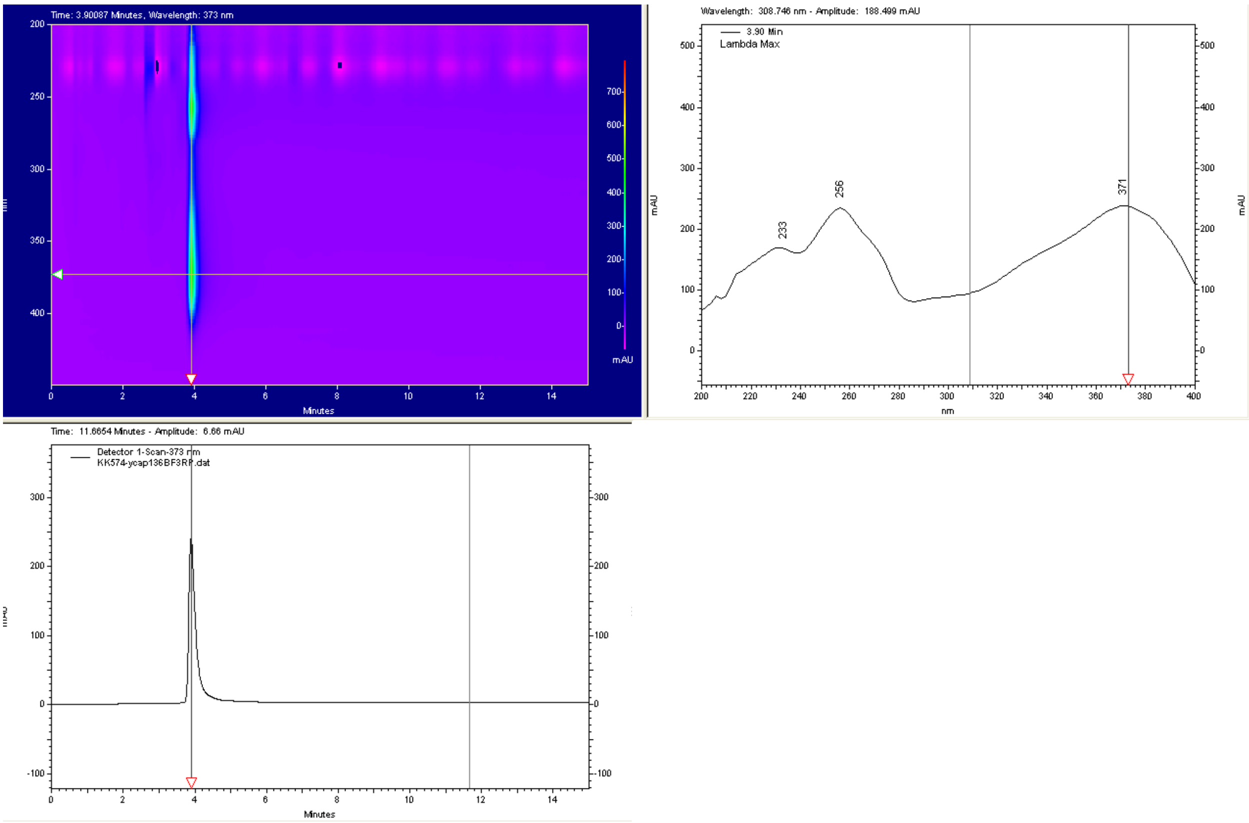

Top left: HPLC profile of UV absorption of probe **Q6**.

Bottom left: HPLC chromatogram at the local absorbance maximum of probe **Q6**.
Top right: UV absorption spectrum of probe **Q6**.

Probe K8

^{1}H / Methanol-*d*4 / 400 MHz

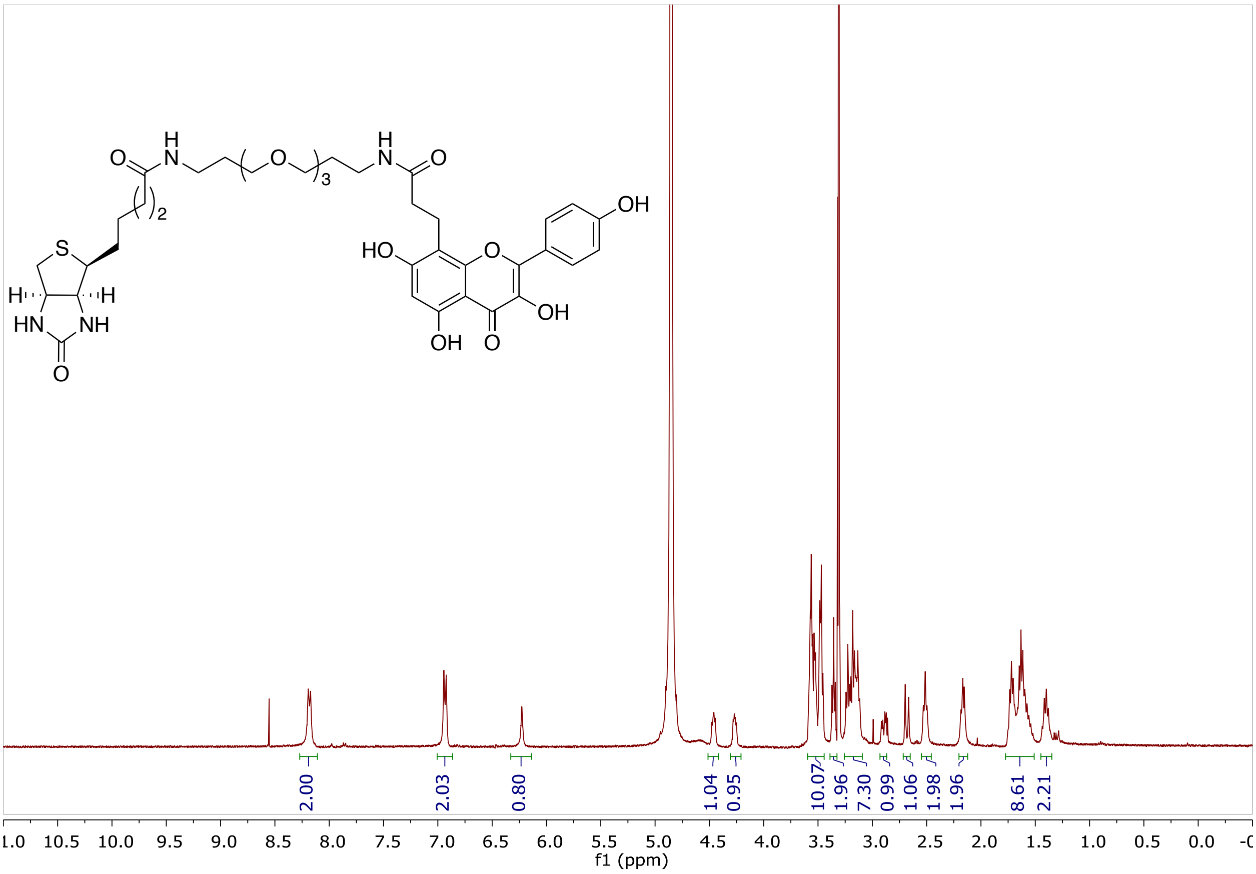

ppm	Hz	Intensity	ppm	Hz	Intensity	ppm	Hz	Intensity
8.55	3423.1	103.1	3.51	1404.4	470.0	2.69	1077.5	394.3
7.72	3089.1	318.3	3.50	1401.5	544.9	2.66	1064.8	300.0
7.63	3055.5	209.5	3.49	1397.9	677.9	2.50	1000.0	293.1
7.63	3053.6	186.2	3.48	1391.7	904.5	2.48	992.6	549.5
7.61	3047.0	219.4	3.46	1385.5	440.7	2.46	985.3	253.8
7.61	3045.1	197.2	3.43	1372.8	396.1	2.19	875.8	259.5
6.88	2752.9	483.2	3.41	1366.6	856.3	2.17	868.3	565.0
6.86	2744.4	468.1	3.40	1360.3	402.2	2.15	860.9	313.1
6.37	2549.1	331.9	3.26	1303.7	391.9	1.74	697.9	400.9
4.47	1788.7	155.6	3.25	1300.8	429.5	1.73	693.8	477.1
4.46	1783.9	175.8	3.24	1297.0	809.7	1.73	691.4	638.7
4.45	1780.7	198.7	3.23	1294.0	777.2	1.72	687.5	662.8
4.44	1776.2	187.5	3.22	1290.3	451.7	1.71	685.1	476.3
4.27	1710.0	209.8	3.22	1287.2	385.0	1.70	681.2	458.7
4.26	1705.6	224.6	3.17	1268.5	117.8	1.69	674.4	249.5
4.25	1702.2	183.1	3.16	1262.8	149.6	1.67	667.4	167.1
4.24	1697.7	173.7	3.15	1259.3	161.2	1.65	660.5	188.9
3.58	1433.5	761.4	3.13	1253.8	176.8	1.63	652.1	259.0
3.57	1430.3	954.8	3.12	1249.4	102.4	1.61	644.5	313.8
3.57	1427.5	967.1	2.96	1184.7	256.5	1.59	636.6	277.9
3.56	1425.2	563.8	2.94	1177.5	533.3	1.57	628.6	175.0
3.54	1417.6	634.9	2.92	1170.0	294.0	1.43	572.9	107.0

3.54	1415.0	464.2	2.91	1164.4	218.6	1.41	565.5	330.1
3.53	1412.0	530.7	2.90	1159.4	215.0	1.39	557.7	363.6
3.52	1408.6	496.3	2.88	1151.6	278.9	1.37	549.9	202.4
3.51	1406.7	543.1	2.87	1146.6	270.5			

^{13}C / Methanol-d_4 / 100 MHz

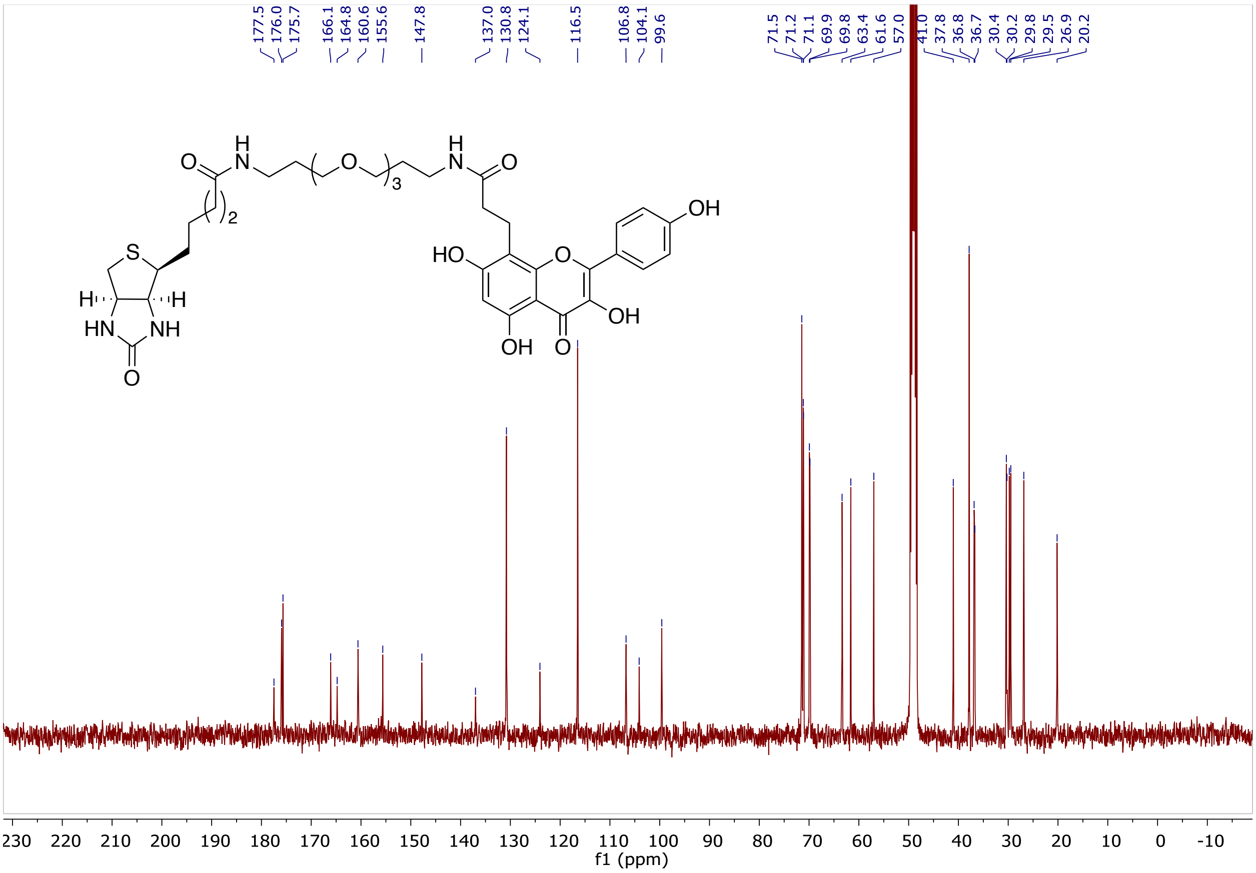

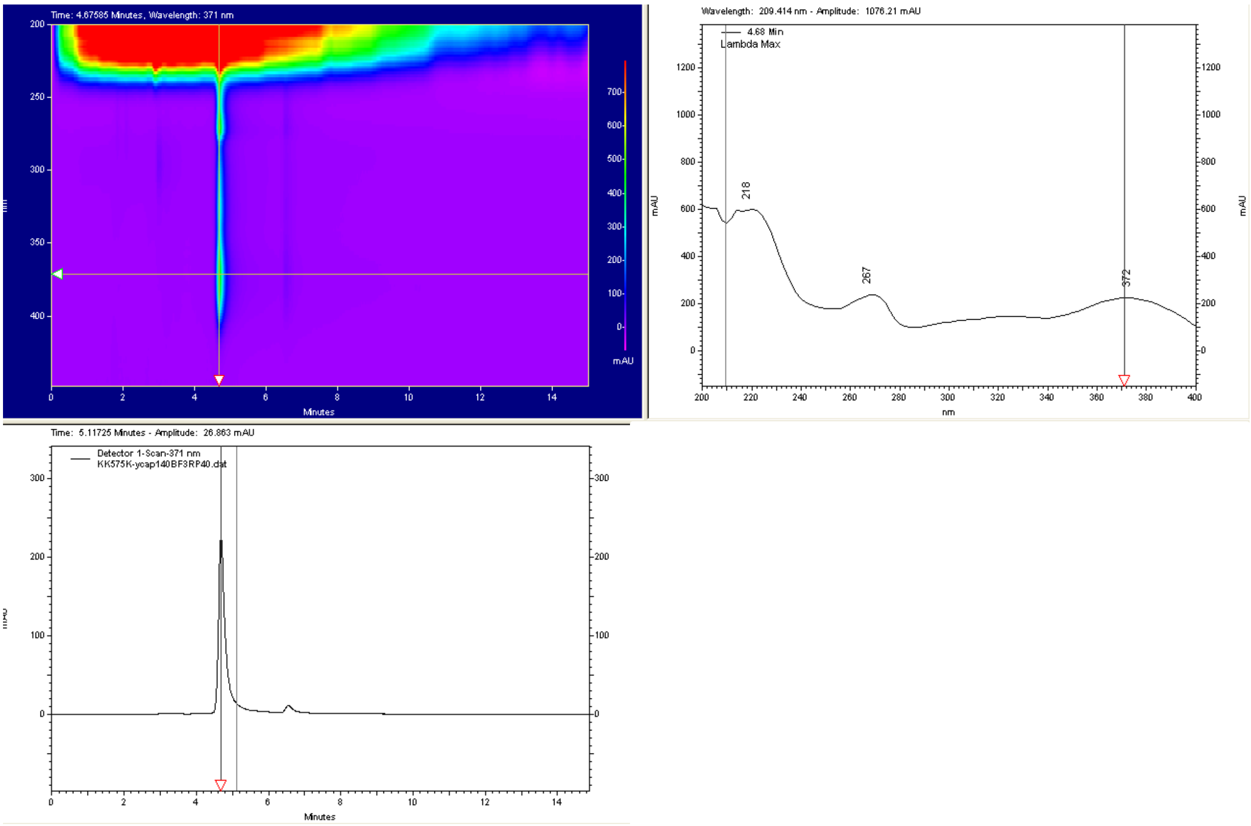

Top left: HPLC profile of UV absorption of probe **K8**.
Bottom left: HPLC chromatogram at the local absorbance maximum of probe **K8**;.
Top right: UV absorption spectrum of probe **K8**.

Annexes

Supplemental information can be found in the following joined Excel documents. Excel documents 1-4 contain the lists of **highly significant proteins**. The name refers firstly to the proteome and secondly to the database used for interrogation. The Excel documents 1 and 2 contain each four worksheets corresponding to the four different assays (see Figure 3 in the main text). The Excel documents 3 and 4 contain each six worksheets corresponding to the six different assays (see Figure 5 in the main text). An additional Excel document 5 contains lists of proteins appearing as highly significant from two assays (**intersections**). The worksheets are named according to the conditions in common for both assays followed by the intersection specifying the two different assays (*e.g.*, O-Q6_Q8 means method **O**, intersection between probe **Q6** and **Q8**; Q8-O_N means probe **Q8**, intersection between methods **O** and **N**). The last Excel document contains the **direct comparison** between the abundances of proteins captured by probes **Q6** and **Q8**.

The Excel documents are:

1) Rudbeckia_Helianthus.xlsx:
   a) Q6_O: 355 proteins
   b) Q8_O: 368 proteins
   c) K8_O: 121 proteins
   d) Q8_N: 402 proteins

2) Rudbeckia_ Rudbeckia.xlsx:
   a) Q6_O: 478 proteins
   b) Q8_O: 497 proteins
   c) K8_O: 168 proteins
   d) Q8_N: 529 proteins

3) Tagetes_Helianthus.xlsx: Q8_O: 28 proteins

4) Tagetes_Tagetes.xlsx: Q8_O: 44 proteins

5) Rudbeckia_intersections.xlsx:
   a) O-Q6_Q8_intersection: 345 proteins
   b) O-K8_Q8_intersection: 118 proteins
   c) Q8-O_N_intersection: 304 proteins

6) Direct_comparison_Q8_vs_Q6.xlsx
